# Sodium assessment in neonates, infants, and children: a systematic review

**DOI:** 10.1007/s00431-022-04543-3

**Published:** 2022-07-12

**Authors:** Antonio Corsello, Sabrina Malandrini, Mario G. Bianchetti, Carlo Agostoni, Barbara Cantoni, Francesco Meani, Pietro B. Faré, Gregorio P. Milani

**Affiliations:** 1 Department of Clinical Sciences and Community Health, Università Degli Studi Di Milano, Milan, Italy; 2 Faculty of Biomedical Sciences, Università Della Svizzera Italiana, Lugano, Switzerland; 3 Pediatric Unit, Fondazione IRCCS Ca’ Granda Ospedale Maggiore Policlinico, Milan, Italy; 4 Department of Gynecology and Obstetrics, Centro Di Senologia Della Svizzera Italiana, Ente Ospedaliero Cantonale, Lugano, Switzerland; 5 Department of Internal Medicine, Ente Ospedaliero Cantonale, 6600 Locarno, Switzerland

**Keywords:** Hyponatremia, Electrolytes, Pediatrics, Laboratory, Potentiometry

## Abstract

Hyponatremia is a common disorder in childhood. The indirect and the direct potentiometry are currently the most popular techniques employed for sodium assessment, although discrepancies between the two techniques may be > 10 mmol/L. It is known that < 20% of the recently published articles report information about the technique used for sodium analysis, but no data are available on pediatric studies. This study aimed at investigating the laboratory technique employed for sodium measurement in studies conducted in childhood. A systematic literature search in PubMed, Embase, and Web of Science was undertaken to identify articles containing the word “hyponatremia” in the title between 2013 and 2020. Papers with < 10 subjects were excluded. A total of 565 articles were included. Information on the laboratory technique used for sodium analysis was more commonly (*p* = 0.035) reported in pediatric (*n* = 15, 28%) than in non-pediatric (*n* = 81, 16%) reports. The frequency of reports with and without information on the technique for sodium assessment was not different with respect to the study characteristics, the quartile of the journal where the paper was published, the country income setting, and the inclusion of neonates among the 54 pediatric studies.

*   Conclusion*: Most pediatric papers do not report any information on the technique used for sodium analysis. Although international authorities have recommended the implementation of direct potentiometry, a low awareness on this issue is still widespread in pediatric research.

**Table:** 

**What is Known:**
• *Direct potentiometry and indirect potentiometry are currently employed for sodium analysis in blood.*
• *Direct potentiometry is more accurate.*
**What is New:**
• *Less than 30% of pediatric articles provide information on the technique employed for sodium analysis in blood.*
• *Indirect potentiometry is more frequently employed than direct potentiometry in pediatric studies.*

**Supplementary Information:**

The online version contains supplementary material available at 10.1007/s00431-022-04543-3.

## Introduction

Hyponatremia is a frequent laboratory finding in childhood and may by complicated by neurological damage [1, 2]. With the gradual abandonment of flame photometry, indirect and direct potentiometry are nowadays the most popular techniques for sodium analysis in clinical practice and research [3].

The indirect potentiometry is usually employed by laboratory analyzers whereas the direct technique by “point-of-care testing” tools such as blood gas analyzers. Although the agreement between the two methods is considered good, the results obtained by indirect potentiometry may be falsely increased or decreased by up to 10 mmol/L [4, 5].

A recent systematic review found that > 80% of the published articles do not report information about the technique used for sodium analysis [6]. However, no similar data are available on pediatric studies. The aim of this analysis of the literature is to investigate if data on the laboratory technique employed for sodium measurement are reported in studies on children, which technique is more frequently used, and if there are differences with studies conducted in adults.

## Methods

### Literature search and study selection

A systematic search of the literature in the National Library of Medicine database (PubMed), Excerpta Medica database (Embase), and Web of Science was undertaken to identify articles containing the word “hyponatremia” or “hyponatraemia” in the title according to the 2020 PRISMA guidelines. Only articles published in English from 2013 to 2020 as full text were retained. Articles including < 10 cases or not conducted in humans were excluded. Two authors independently screened the articles for eligibility.

### Data extraction and study classification

From retained articles, the following information was extracted: (1) population type (pediatric vs non pediatric), (2) number of included subjects, (3) laboratory technique employed for sodium assessment (flame photometry, direct potentiometry, indirect potentiometry, not reported), (4) data collection (retrospective vs prospective), (5) study design (observational vs interventional), (6) quartile of the journal where the paper was published, (7) country income setting (high versus other). Pediatric studies were also subdivided in those with and without neonates.

The Journal Citation Reports (Clarivate Analytics’ Web of Science™) was used to assign the quartile ranking journal where the paper was published, in the year of publication. If the journal was listed in more than one subject area, the highest quartile was chosen.

### Outcomes

For the present study, the primary outcome was the percentage of pediatric papers reporting information on the technique used for sodium assessment. The secondary outcomes were (1) the percentage of the non-pediatric papers reporting information on the technique used for sodium assessment and (2) the characteristics of the papers reporting the information on the technique used for sodium assessment.

### Data management and statistical analysis

Corresponding authors of each article with no information about the technique employed for sodium measurement were contacted to gather the information. A reminder was sent to non-responders 1.5–2 months after the first email. To manage missing data, pairwise deletion was used. For data analysis, the two-tailed Fisher exact test was employed to compare categorical data, and statistical significance was set at *p* < 0.05. Prism 9 was used for the analysis.

## Results

### Search results

**Fig. 1:**
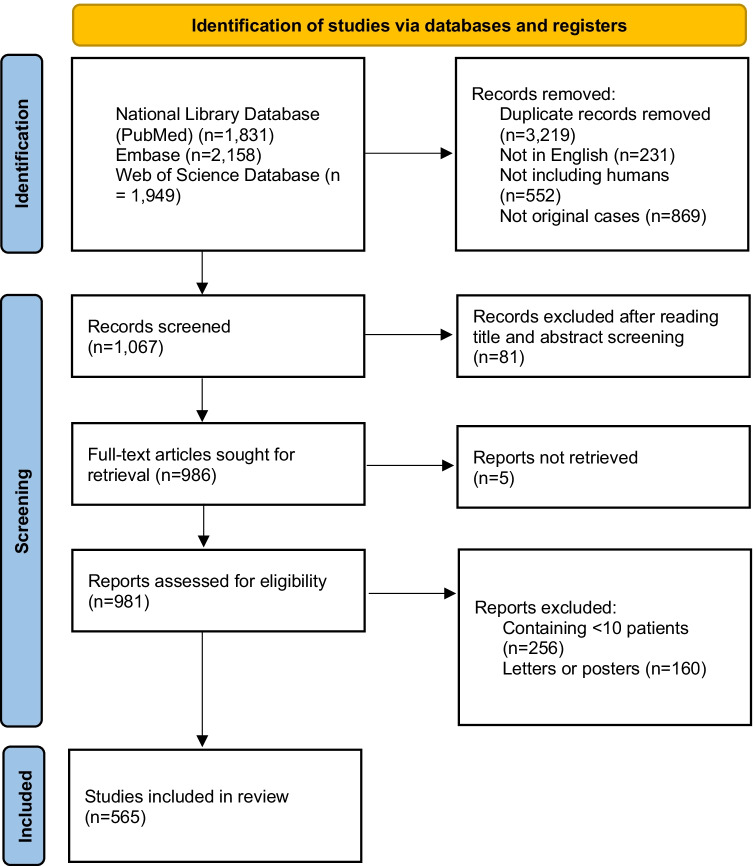
Flowchart of the literature search process

The literature search process is reported in Fig. 1 and the full list of included papers is given in the supplementary online material (pediatric studies in supplementary file 1 and non-pediatric studies in supplementary file 2). Five hundred and sixty-five articles were included. They were published from the following continents: 225 from Asia (Japan, *n* = 46; India, *n* = 35; Korea, *n* = 33; China, *n* = 29; Pakistan, *n* = 19; Turkey, *n* = 16; Taiwan, *n* = 15; Israel, *n* = 12; Iran, *n* = 6; Saudi Arabia, *n* = 4; Indonesia, *n* = 2; Singapore, *n* = 2; Bangladesh, *n* = 1; Nepal, *n* = 1; Philippines, *n* = 1; Russia, *n* = 1; Thailand, *n* = 1; Vietnam, *n* = 1), 162 from Europe (Italy, *n* = 30; Spain, *n* = 19; Germany, *n* = 17; Switzerland, *n* = 14; Denmark, *n* = 13; Sweden, *n* = 11; France, *n* = 9; Belgium, *n* = 8; Netherlands, *n* = 8; Poland, *n* = 8; Austria, *n* = 4; Ireland, *n* = 4; UK, *n* = 4; Czech Republic, *n* = 3; Greece, *n* = 2; Finland, *n* = 2; Romania, *n* = 2; Croatia, *n* = 1; Norway, *n* = 1; Portugal, *n* = 1; Serbia, *n* = 1), 154 from North America (USA, *n* = 140; Canada, *n* = 14), 9 from South America (Brazil, *n* = 5; Argentina, *n* = 3; Mexico *n* = 1), 9 from Oceania (Australia, *n* = 8; New Zealand, *n* = 1), and 6 from Africa (Egypt, *n* = 2; Ethiopia, *n* = 1; Nigeria, *n* = 1; South Africa, *n* = 1; Tunisia, *n* = 1).

### Findings

Table 1 reports the characteristics of the 565 included articles. Pediatric and non-pediatric studies did not significantly differ with respect to number of subjects per publication, study design, quartile of the journal, and country income setting. Information on the laboratory technique employed for sodium assessment was more commonly (*p* = 0.034) reported in pediatric (*n* = 15, 28%) than in non-pediatric (*n* = 81, 16%) reports.

**Table 1:** Characteristics of the 565 articles included in this analysis

		**Pediatric**	**Non pediatric**	***p*-value**
**Publications, *n***		54	511	
**Study classification**				
	Observational, *n* (%)	45 (83)	396 (77)	
	Interventional, n (%)	9 (17)	115 (23)	0.389
**Data collection**				
	Prospective, *n* (%)	19 (35)	194 (38)	
	Retrospective, *n* (%)	35 (65)	317 (62)	0.769
**Subjects per publication**				
	10–99, *n* (%)	15 (28)	143 (28)	
	≥ 100, *n* (%)	39 (72)	368 (72)	1.000
**Journal impact factor quartile**				
	First quartile, *n* (%)	10 (19)	152 (30)	
	Further quartiles/no impact factor, *n* (%)	44 (81)	359 (70)	0.112
**Countries by income**				
	High-income countries, *n* (%)	37 (69)	382 (75)	
	Middle- or low-income countries, *n* (%)	17 (31)	129 (25)	0.329
**Na**^**+**^** determination–laboratory technique**				
	Information published, *n* (%)	15 (28)	81 (16)	
	Information after inquiry/unavailable, *n* (%)	39 (72)	430 (84)	0.035

Information on the technique employed for sodium assessment was obtained from the corresponding authors for further 175 reports. Indirect potentiometry was the most used technique (81%) among both pediatric and non-pediatric studies (Table 2). In children, however, a larger (*p* = 0.001) subset of studies was performed using the direct method (45% versus 15%).

**Table 2:** Relative frequency of direct and indirect potentiometry for determination of sodium in pediatric and non-pediatric reports (information provided either from the publication or after inquiry). Both indirect and direct potentiometry were concurrently used in 29 reports (pediatric, *n* = 1), flame spectrometry in two non-pediatric reports

	**All**	**Pediatric**	**Non-pediatric**	***p*-value**
Indirect, *n* (%)	197 (81)	16 (57)	181 (85)	
Direct, *n* (%)	45 (19)	12 (45)	33 (15)	0.001

**Fig. 2:**
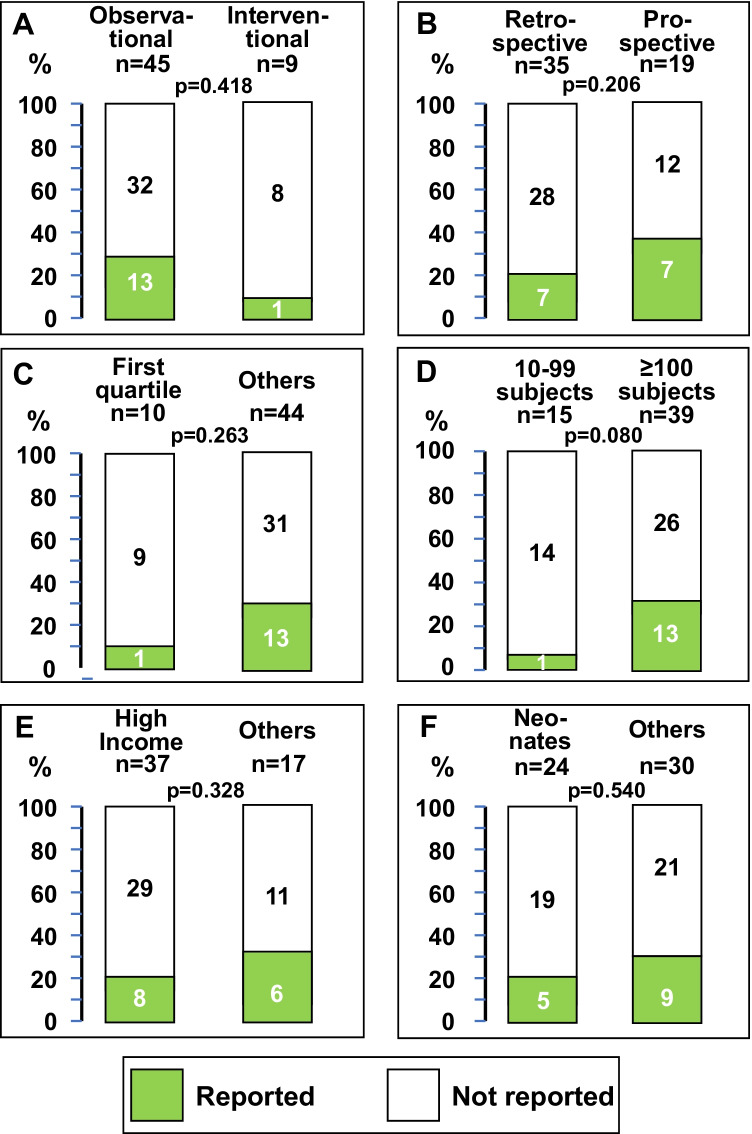
Pediatric studies reporting within the text, the information on the technique employed for sodium assessment. Studies are compared for **A** design, **B** data collection, **C** journal quartile ranking, **D** number of included subjects, **E** country income setting, and **F** inclusion of neonates

The frequency of reports with and without information on the technique for sodium assessment was not different with respect to study design (observational vs interventional), the data collection (retrospective vs prospective), the quartile of the journal where the paper was published (first vs others), the number of included subjects (10–99 vs ≥ 100), the country income setting (high versus others), and the inclusion of neonates (yes vs no) among the 54 pediatric studies (Fig. 2).

## Discussion

This is the first systematic analysis of the literature on the techniques employed to assess sodium in childhood. This study points out that more than about two-third of the papers dealing with hyponatremia in pediatrics do not report any information on the technique used to assess sodium. On the other hand, the information was more frequently available in pediatric than in non-pediatric studies. Moreover, indirect potentiometry was overall the most employed technique.

Most pediatric articles (> 70%) included in our analysis fail to report any information on the technique employed for sodium analysis, independently from the study characteristics (including the involvement of neonates), the journal quartile ranking, and the country income setting.

This widespread attitude suggests that the techniques are considered interchangeable by most pediatric (and non-pediatric) researchers. This speculation is also supported by the fact that even systematic reviews and meta-analyses about the effects of medical interventions on sodium levels include data without considering the technique used to measure this electrolyte in the original studies [7–10]. This choice has a potential high relevance, considering that some of these papers included also critically ill patients: in this setting, the discrepancy between the two methods is often clinically relevant, i.e., > 4 mmol/L [3, 11].

The findings obtained in the original papers or after inquiry about the technique used for sodium analysis deserve some further considerations. Interestingly, pediatric studies reported such information more often than non-pediatric studies (28% vs 16%, respectively). Moreover, although indirect potentiometry represents the most frequently used technique, the direct potentiometry is more frequently employed in pediatric (45%) than in non-pediatric studies (15%). Indirect potentiometry, which utilizes diluted blood samples, assumes that the non-watery fraction of plasma is ≈7% [12]. A reduction in lipid and protein concentration might result in an increase of the plasma watery fraction and, consequently, possible spuriously high sodium measurements [12, 13]. On the other hand, direct potentiometry, which utilizes undiluted blood samples, is unaffected by changes of the non-watery fraction of plasma [4]. Therefore, the use of direct potentiometry is especially important for sodium assessment in neonates and infants, who typically present with lower circulating levels of immunoglobulins, clotting and anti-clotting factors, and lipids [14, 15].

Sodium abnormalities in childhood might occur both at presentation and during hospitalization [16, 17]. Neurological injuries and death have been reported due to hospital acquired-dysnatremia [18, 19]. Therefore, sodium monitoring is essential, and the combination of different sampling and analytical techniques might result in suboptimal children management [20–22]. In addition, direct potentiometry requires small amount of blood and has a relevantly shorter turnaround time [6, 13]. Therefore, most international societies recommend that indirect technique is progressively abandoned [23, 24].

This study has some important implications. First, it points out that the possibility to compare or pool data from available literature dealing with hyponatremia is affected by the unavailability of information on the technique for sodium measurement. Second, a greater awareness among pediatric researchers on the potential discrepancies between the two techniques is needed. Third, our findings suggest that also pediatric hospitalists poorly consider the technique used for sodium assessment. Finally, we suggest that the description of the technique for sodium assessment should be included in every scientific report and considered to rate the study quality.

This analysis has at least two limitations. Firstly, it includes only papers with the term “hyponatremia” in the title. Therefore, it does not consider papers which assessed or monitored sodium levels as secondary outcomes. This strategy could have led to underestimate the issue of sodium measurement in the pediatric literature, since included papers had a special emphasis on sodium and might be more prone to report information on its measurement. Moreover, papers focusing on hypernatremia were not included. However, hypernatremia is nowadays rather rare in childhood [25, 26].

## Conclusions

This analysis points out that most reports (adult papers more often than pediatric papers) do not report any information on the technique used to assess sodium and, when reported, indirect potentiometry is still the most popular technique. Although international authorities have recommended the implementation of direct potentiometry, a low awareness on this issue is still widespread in pediatric research.

## Supplementary Information

Below is the link to the electronic supplementary material.

Supplementary file1 (DOCX 19 KB)

Supplementary file2 (DOCX 82 KB)

## Data Availability

Upon reasonable request to the corresponding author.
