# Supplementary material for: Sodium assessment in neonates, infants, and children: a systematic review
Source: Eur J Pediatr. 2022 Jul 12;181(9):3413–9. doi: 10.1007/s00431-022-04543-3 (PMC9395449; doi:10.1007/s00431-022-04543-3)
Supplement: Supplementary file 1 — Supplementary file1 (DOCX 19 KB) [file 431_2022_4543_MOESM1_ESM.docx]

**Pediatric studies**

1. Adachi M, Takamasu T, Inuo C (2019) Hyponatremia secondary to severe atopic dermatitis in early infancy. Pediatr Int 61:544–550. https://doi.org/10.1111/ped.13865

2. Al-Sofyani KA (2019) Prevalence and Clinical Significance of Hyponatremia in Pediatric Intensive Care. J Pediatr Intensive Care 8:130–137. https://doi.org/10.1055/s-0038-1676635

3. Alconcher LF, Coccia PA, Suarez ADC, et al (2018) Hyponatremia: a new predictor of mortality in patients with Shiga toxin-producing Escherichia coli hemolytic uremic syndrome. Pediatr Nephrol 33:1791–1798. https://doi.org/10.1007/s00467-018-3991-6

4. Belzer JS, Williams CN, Riva-Cambrin J, et al (2014) Timing, duration, and severity of hyponatremia following pediatric brain tumor surgery*. Pediatr Crit Care Med 15:456–463. https://doi.org/10.1097/PCC.0000000000000154

5. Bezinover D, Nahouraii L, Sviatchenko A, et al (2020) Hyponatremia Is Associated With Increased Mortality in Children on the Waiting List for Liver Transplantation. Transplant Direct 6:e604. https://doi.org/10.1097/TXD.0000000000001050

6. Bibi S, Bibi S, Gilani SYH, et al (2015) FREQUENCY OF HOSPITAL ACQUIRED HYPONATREMIA IN A PEDIATRIC TERTIARY CARE SETTING. J Ayub Med Coll Abbottabad 27:560–563

7. Davalos MC, Barrett R, Seshadri S, et al (2013) Hyponatremia during arginine vasopressin therapy in children following cardiac surgery. Pediatr Crit Care Med 14:290–297. https://doi.org/10.1097/PCC.0b013e3182720473

8. Flores Robles CM, Cuello García CA (2016) A prospective trial comparing isotonic with hypotonic maintenance fluids for prevention of hospital-acquired hyponatraemia. Paediatr Int Child Health 36:168–174. https://doi.org/10.1179/2046905515Y.0000000047

9. Gokçe İK, Oguz SS (2020) Late onset hyponatremia in preterm newborns: is the sodium content of human milk fortifier insufficient? J Matern Fetal Neonatal Med 33:1197–1202. https://doi.org/10.1080/14767058.2018.1517314

10. Hao TK (2019) Prevalence and Risk Factors for Hyponatremia in Preterm Infants. Open Access Maced J Med Sci 7:3201–3204. https://doi.org/10.3889/oamjms.2019.558

11. Haseeb M, Engade M, Valecha A, et al (2019) Hyponatremia in Children Hospitalised with Community-acquired Pneumonia: A prospective observational study. J Clin Diagn Res 13:SC01–SC04. https://doi.org/10.7860/JCDR/2019/42103.13238

12. Hasegawa K, Stevenson MD, Mansbach JM, et al (2015) Association Between Hyponatremia and Higher Bronchiolitis Severity Among Children in the ICU With Bronchiolitis. Hosp Pediatr 5:385–389. https://doi.org/10.1542/hpeds.2015-0022

13. Inamdar P, Masavkar S, Shanbag P (2016) Hyponatremia in children with tuberculous meningitis: A hospital-based cohort study. J Pediatr Neurosci 11:182–187. https://doi.org/10.4103/1817-1745.193376

14. Jamil M, Khan A, Faraz M, et al (2021) Frequency of hyponatremia in children with pneumonia. Pak J Med Health Sci 15:742–744

15. Janczar S, Zalewska-Szewczyk B, Mlynarski W (2017) Severe Hyponatremia in a Single-Center Series of 84 Homogenously Treated Children With Acute Lymphoblastic Leukemia. J Pediatr Hematol Oncol 39:e54–e58. https://doi.org/10.1097/MPH.0000000000000758

16. Karageorgos SA, Kratimenos P, Landicho A, et al (2018) Hospital-Acquired Hyponatremia in Children Following Hypotonic versus Isotonic Intravenous Fluids Infusion. Children (Basel) 5:E139. https://doi.org/10.3390/children5100139

17. Karapınar DY, Şahin A, Özen S, et al (2020) Hyponatremia in children with acute lymphoblastic leukemia. J Pediat Res 7:139–145. https://doi.org/10.4274/JPR.GALENOS.2019.46547

18. Kim Y-J, Lee JA, Oh S, et al (2015) Risk factors for late-onset hyponatremia and its influence on neonatal outcomes in preterm infants. J Korean Med Sci 30:456–462. https://doi.org/10.3346/jkms.2015.30.4.456

19. Kishimoto K, Kobayashi R, Sano H, et al (2016) Analysis of Risk Factors for Hyponatremia During or Following Chemotherapy in Children With Cancer: A Hospital-based, Retrospective Cohort Study. J Pediatr Hematol Oncol 38:443–448. https://doi.org/10.1097/MPH.0000000000000478

20. Luu R, DeWitt PE, Reiter PD, et al (2013) Hyponatremia in children with bronchiolitis admitted to the pediatric intensive care unit is associated with worse outcomes. J Pediatr 163:1652-1656.e1. https://doi.org/10.1016/j.jpeds.2013.06.041

21. Mazzoni MB, Milani GP, Bernardi S, et al (2019) Hyponatremia in infants with community-acquired infections on hospital admission. PLoS One 14:e0219299. https://doi.org/10.1371/journal.pone.0219299

22. Milani GP, Rocchi A, Teatini T, et al (2017) Hyponatremia in infants with new onset moderate-severe bronchiolitis: A cross-sectional study. Respir Med 133:48–50. https://doi.org/10.1016/j.rmed.2017.10.028

23. Miura K, Harita Y, Takahashi N, et al (2020) Nonosmotic secretion of arginine vasopressin and salt loss in hyponatremia in Kawasaki disease. Pediatr Int 62:363–370. https://doi.org/10.1111/ped.14036

24. Navaeifar MR, Abbaskhanian A, Farmanbarborji A (2020) Relation between Febrile Seizure Recurrence and Hyponatremia in Children: A Single-center Trial. Journal of pediatric neurosciences. https://doi.org/10.4103/jpn.JPN_4_19

25. Omoifo CE, Edomwonyi NP, Idogun SE (2018) Incidence of Hyponatraemia Following the Use of Three Different Intravenous Fluids in Paediatric Surgery. Afr J Paediatr Surg 15:69–72. https://doi.org/10.4103/ajps.AJPS_40_16

26. Pappo A, Gavish R, Goldberg O, et al (2021) Hyponatremia in childhood urinary tract infection. Eur J Pediatr 180:861–867. https://doi.org/10.1007/s00431-020-03808-z

27. Park JS, Jeong S-A, Cho JY, et al (2020) Risk Factors and Effects of Severe Late-Onset Hyponatremia on Long-Term Growth of Prematurely Born Infants. Pediatr Gastroenterol Hepatol Nutr 23:472–483. https://doi.org/10.5223/pghn.2020.23.5.472

28. Park SW, Shin SM, Jeong M, et al (2018) Hyponatremia in children with respiratory infections: a cross-sectional analysis of a cohort of 3938 patients. Sci Rep 8:16494. https://doi.org/10.1038/s41598-018-34703-1

29. Pemde HK, Dutta AK, Sodani R, Mishra K (2015) Isotonic intravenous maintenance fluid reduces hospital acquired hyponatremia in young children with central nervous system infections. Indian J Pediatr 82:13–18. https://doi.org/10.1007/s12098-014-1436-1

30. Poddighe D (2016) Common finding of mild hyponatremia in children evaluated at the Emergency Department and its correlation with plasma C-reactive protein values. Minerva Pediatr 68:173–176

31. Price JF, Kantor PF, Shaddy RE, et al (2016) Incidence, Severity, and Association With Adverse Outcome of Hyponatremia in Children Hospitalized With Heart Failure. Am J Cardiol 118:1006–1010. https://doi.org/10.1016/j.amjcard.2016.07.014

32. S K R, Dakshayani B, R P (2017) Full Volume Isotonic (0.9%) vs. Two-Thirds Volume Hypotonic (0.18%) Intravenous Maintenance Fluids in Preventing Hyponatremia in Children Admitted to Pediatric Intensive Care Unit-A Randomized Controlled Study. J Trop Pediatr 63:454–460. https://doi.org/10.1093/tropej/fmx012

33. Sachdev A, Pandharikar N, Gupta D, et al (2017) Hospital-acquired Hyponatremia in Pediatric Intensive Care Unit. Indian J Crit Care Med 21:599–603. https://doi.org/10.4103/ijccm.IJCCM_131_17

34. Saldarriaga C, Lyssikatos C, Belyavskaya E, et al (2018) Postoperative Diabetes Insipidus and Hyponatremia in Children after Transsphenoidal Surgery for Adrenocorticotropin Hormone and Growth Hormone Secreting Adenomas. J Pediatr 195:169-174.e1. https://doi.org/10.1016/j.jpeds.2017.11.042

35. Schuster JE, Palac HL, Innocentini N, et al (2017) Hyponatremia Is a Feature of Kawasaki Disease Shock Syndrome: A Case-Control Study. J Pediatric Infect Dis Soc 6:386–388. https://doi.org/10.1093/jpids/piw081

36. Shahrin L, Chisti MJ, Huq S, et al (2016) Clinical Manifestations of Hyponatremia and Hypernatremia in Under-Five Diarrheal Children in a Diarrhea Hospital. J Trop Pediatr 62:206–212. https://doi.org/10.1093/tropej/fmv100

37. Sharma R, Stein D (2014) Hyponatremia after desmopressin (DDAVP) use in pediatric patients with bleeding disorders undergoing surgeries. J Pediatr Hematol Oncol 36:e371-375. https://doi.org/10.1097/MPH.0000000000000185

38. Shein SL, Slain K, Martinez Schlurmann N, et al (2017) Hyponatremia and Hypotonic Intravenous Fluids Are Associated With Unfavorable Outcomes of Bronchiolitis Admissions. Hosp Pediatr 7:263–270. https://doi.org/10.1542/hpeds.2016-0205

39. Shima R, Sawano K, Shibata N, et al (2020) Timing of hyponatremia development in patients with salt-wasting-type 21-hydroxylase deficiency. Clin Pediatr Endocrinol 29:105–110. https://doi.org/10.1297/cpe.29.105

40. Shukla S, Basu S, Moritz ML (2016) Use of Hypotonic Maintenance Intravenous Fluids and Hospital-Acquired Hyponatremia Remain Common in Children Admitted to a General Pediatric Ward. Front Pediatr 4:90. https://doi.org/10.3389/fped.2016.00090

41. Silva Duarte Dos Santos R, Kieling CO, Adami MR, et al (2020) Hypervolemic hyponatremia and transplant-free survival in children with cirrhosis due to biliary atresia. Pediatr Transplant 24:e13687. https://doi.org/10.1111/petr.13687

42. Sorkhi H, Salehi Omran MR, Barari Savadkoohi R, et al (2013) CSWS Versus SIADH as the Probable Causes of Hyponatremia in Children With Acute CNS Disorders. Iran J Child Neurol 7:34–39

43. Späth C, Sjöström ES, Ahlsson F, et al (2017) Sodium supply influences plasma sodium concentration and the risks of hyper- and hyponatremia in extremely preterm infants. Pediatr Res 81:455–460. https://doi.org/10.1038/pr.2016.264

44. Stephens K, Miller JL, Lewis TV, et al (2020) Hyponatremia With Intravenous Sulfamethoxazole/Trimethoprim in Children. Ann Pharmacother 54:351–358. https://doi.org/10.1177/1060028019887919

45. Storey C, Dauger S, Deschenes G, et al (2019) Hyponatremia in children under 100 days old: incidence and etiologies. Eur J Pediatr 178:1353–1361. https://doi.org/10.1007/s00431-019-03406-8

46. Tagarro A, Martín M-D, Del-Amo N, et al (2018) Hyponatremia in children with pneumonia rarely means SIADH. Paediatr Child Health 23:e126–e133. https://doi.org/10.1093/pch/pxy003

47. Turner II, Ruzmetov M, Niu J, et al (2021) Scavenging right atrial Bretschneider histidine-tryptophan-ketoglutarate cardioplegia: Impact on hyponatremia and seizures in pediatric cardiac surgery patients. The Journal of Thoracic and Cardiovascular Surgery 162:228–237. https://doi.org/10.1016/j.jtcvs.2020.08.098

48. Velasco P, Alcaraz AJ, Oikonomopoulou N, et al (2018) Hospital-acquired hyponatremia: Does the type of fluid therapy affect children admitted to intensive care? Rev Chil Pediatr 89:42–50. https://doi.org/10.4067/S0370-41062018000100042

49. Williams CN, Belzer JS, Riva-Cambrin J, et al (2014) The incidence of postoperative hyponatremia and associated neurological sequelae in children with intracranial neoplasms. J Neurosurg Pediatr 13:283–290. https://doi.org/10.3171/2013.12.PEDS13364

50. Williams CN, Riva-Cambrin J, Bratton SL (2016) Etiology of postoperative hyponatremia following pediatric intracranial tumor surgery. J Neurosurg Pediatr 17:303–309. https://doi.org/10.3171/2015.7.PEDS15277

51. Williams CN, Riva-Cambrin J, Presson AP, Bratton SL (2015) Hyponatremia and poor cognitive outcome following pediatric brain tumor surgery. J Neurosurg Pediatr 15:480–487. https://doi.org/10.3171/2014.10.PEDS14368

52. Wrotek A, Jackowska T (2013) Hyponatremia in children hospitalized due to pneumonia. Adv Exp Med Biol 788:103–108. https://doi.org/10.1007/978-94-007-6627-3_16

53. Yang S-C, Wang C-H, Chen C-L, et al (2014) Acquired Hyponatremia in Pediatric Living Donor Liver Transplantation. Ann Transplant 19:609–613. https://doi.org/10.12659/AOT.892191

54. Zheng F, Ye X, Shi X, et al (2019) Hyponatremia in Children With Bacterial Meningitis. Front Neurol 10:421. https://doi.org/10.3389/fneur.2019.00421
