# Supplementary material for: Sodium assessment in neonates, infants, and children: a systematic review
Source: Eur J Pediatr. 2022 Jul 12;181(9):3413–9. doi: 10.1007/s00431-022-04543-3 (PMC9395449; doi:10.1007/s00431-022-04543-3)
Supplement: Supplementary file 2 — Supplementary file2 (DOCX 82 KB) [file 431_2022_4543_MOESM2_ESM.docx]

**Non-pediatric studies**

1. Abola MV, Tanenbaum JE, Bomberger TT, et al (2019) Preoperative Hyponatremia Is Associated with Reoperation and Prolonged Length of Hospital Stay following Total Knee Arthroplasty. J Knee Surg 32:344–351. https://doi.org/10.1055/s-0038-1641156
2. Achinger SG, Arieff AI, Kalantar-Zadeh K, Ayus JC (2014) Desmopressin acetate (DDAVP)-associated hyponatremia and brain damage: a case series. Nephrol Dial Transplant 29:2310–2315. https://doi.org/10.1093/ndt/gfu263
3. Adams AL, Li BH, Bhandari S, et al (2019) Chronic hyponatremia and association with osteoporosis among a large racially/ethnically diverse population. Osteoporos Int 30:853–861. https://doi.org/10.1007/s00198-018-04832-4
4. Afshinnia F, Sundaram B, Ackermann RJ, Wong KK (2015) Hyponatremia and osteoporosis: reappraisal of a novel association. Osteoporos Int 26:2291–2298. https://doi.org/10.1007/s00198-015-3108-z
5. Ahluwalia V, Wade JB, Thacker L, et al (2013) Differential impact of hyponatremia and hepatic encephalopathy on health-related quality of life and brain metabolite abnormalities in cirrhosis. J Hepatol 59:467–473. https://doi.org/10.1016/j.jhep.2013.04.023
6. Aicale R, Tarantino D, Maffulli N (2017) Prevalence of Hyponatremia in Elderly Patients with Hip Fractures: A Two-Year Study. Med Princ Pract 26:451–455. https://doi.org/10.1159/000480294
7. Al Qahtani M, Alshahrani A, Alskaini A, et al (2013) Prevalence of hyponatremia among patients who used indapamide and hydrochlorothiazide: a single center retrospective study. Saudi J Kidney Dis Transpl 24:281–285. https://doi.org/10.4103/1319-2442.109574
8. Albabtain M, Brenner MJ, Nicklas JM, et al (2016) Hyponatremia, Cognitive Function, and Mobility in an Outpatient Heart Failure Population. Med Sci Monit 22:4978–4985. https://doi.org/10.12659/msm.898538
9. Alem MM (2020) Predictors of Mortality in Patients with Chronic Heart Failure: Is Hyponatremia a Useful Clinical Biomarker? Int J Gen Med 13:407–417. https://doi.org/10.2147/IJGM.S260256
10. Ali K, Workicho A, Gudina EK (2016) Hyponatremia in patients hospitalized with heart failure: a condition often overlooked in low-income settings. Int J Gen Med 9:267–273. https://doi.org/10.2147/IJGM.S110872
11. Almas A, Ahmed N, Khawaja F, Khan AH (2014) Diuretic induced hyponatremia in hypertensive patients. J Coll Physicians Surg Pak 24:606–608. https://doi.org/08.2014/JCPSP.606608
12. Amin A, Deitelzweig S, Christian R, et al (2013) Healthcare resource burden associated with hyponatremia among patients hospitalized for heart failure in the US. J Med Econ 16:415–420. https://doi.org/10.3111/13696998.2013.766615
13. Amin AN, Ortendahl JD, Harmon AL, et al (2019) Costs associated with unplanned readmissions among patients with heart failure with and without hyponatremia. Am J Health Syst Pharm 76:374–380. https://doi.org/10.1093/ajhp/zxy064
14. Annoni F, Fontana V, Brimioulle S, et al (2017) Early Effects of Enteral Urea on Intracranial Pressure in Patients With Acute Brain Injury and Hyponatremia. J Neurosurg Anesthesiol 29:400–405. https://doi.org/10.1097/ANA.0000000000000340
15. Arakawa Y, Shirai Y, Hayashi K, et al (2018) Effects of gene polymorphisms on the risk of severe hyponatremia during DCF chemotherapy for patients with esophageal squamous cell carcinoma. Oncol Lett 16:5455–5462. https://doi.org/10.3892/ol.2018.9236
16. Arakawa Y, Tamura M, Sakuyama T, et al (2015) Early measurement of urinary N-acetyl-β-glucosaminidase helps predict severe hyponatremia associated with cisplatin-containing chemotherapy. J Infect Chemother 21:502–506. https://doi.org/10.1016/j.jiac.2015.03.008
17. Arampatzis S, Gaetcke L-M, Funk G-C, et al (2013) Diuretic-induced hyponatremia and osteoporotic fractures in patients admitted to the emergency department. Maturitas 75:81–86. https://doi.org/10.1016/j.maturitas.2013.02.007
18. Arao K, Fujiwara T, Sakakura K, et al (2013) Hyponatremia as a predictor for worsening heart failure in patients receiving cardiac resynchronization therapy. Circ J 77:116–122. https://doi.org/10.1253/circj.cj-12-0672
19. Aratani S, Hara M, Nagahama M, et al (2017) A low initial serum sodium level is associated with an increased risk of overcorrection in patients with chronic profound hyponatremia: a retrospective cohort analysis. BMC Nephrol 18:316. https://doi.org/10.1186/s12882-017-0732-1
20. Arévalo Lorido JC, Carretero Gómez J, Formiga F, et al (2013) Hyponatremia as predictor of worse outcome in real world patients admitted with acute heart failure. Cardiol J 20:506–512. https://doi.org/10.5603/CJ.2013.0136
21. Arévalo-Lorido JC, Carretero-Gómez J, Robles NR, et al (2019) Prognostic Role of Hyponatremia in Heart Failure Patients Depending on Renal Disease: Clinical Evidence. Cardiology 144:1–8. https://doi.org/10.1159/000502566
22. Arima H, Goto K, Motozawa T, et al (2021) Open-label, multicenter, dose-titration study to determine the efficacy and safety of tolvaptan in Japanese patients with hyponatremia secondary to syndrome of inappropriate secretion of antidiuretic hormone. Endocr J 68:17–29. https://doi.org/10.1507/endocrj.EJ20-0216
23. Arnaoutis G, Anastasiou CA, Suh H, et al (2020) Exercise-Associated Hyponatremia during the Olympus Marathon Ultra-Endurance Trail Run. Nutrients 12:E997. https://doi.org/10.3390/nu12040997
24. Assen A Abouem D, Vandergheynst F, Nguyen T, et al (2014) Hyponatremia at the Emergency Department: a case-control study. Minerva Anestesiol 80:419–428
25. Astaf’eva LI, Kutin MA, Mazerkina NA, et al (2016) [The rate of hyponatremia in neurosurgical patients (comparison between the data from the Burdenko Neurosurgical Instutite and the literature) and recommendations for the diagnosis and treatment]. Zh Vopr Neirokhir Im N N Burdenko 80:57–70. https://doi.org/10.17116/neiro201680157-70
26. Aydinli B, Bolukbasi D, Demir A, et al (2019) Hyponatremia and hypoalbuminemia are predictors of morbi-mortality in coronary artery bypass graft surgery. Kuwait Med J 51:151–156
27. Ayus JC, Fuentes NA, Negri AL, et al (2016) Mild prolonged chronic hyponatremia and risk of hip fracture in the elderly. Nephrol Dial Transplant 31:1662–1669. https://doi.org/10.1093/ndt/gfw029
28. Babaliche P, Madnani S, Kamat S (2017) Clinical Profile of Patients Admitted with Hyponatremia in the Medical Intensive Care Unit. Indian J Crit Care Med 21:819–824. https://doi.org/10.4103/ijccm.IJCCM_257_17
29. Bae MH, Kim JH, Jang SY, et al (2017) Hyponatremia at discharge as a predictor of 12-month clinical outcomes in hospital survivors after acute myocardial infarction. Heart Vessels 32:126–133. https://doi.org/10.1007/s00380-016-0854-6
30. Baek SH, Jo YH, Ahn S, et al (2021) Risk of Overcorrection in Rapid Intermittent Bolus vs Slow Continuous Infusion Therapies of Hypertonic Saline for Patients With Symptomatic Hyponatremia: The SALSA Randomized Clinical Trial. JAMA Intern Med 181:81–92. https://doi.org/10.1001/jamainternmed.2020.5519
31. Baek SH, Kim S, Na KY, et al (2018) Predialysis hyponatremia and mortality in elderly patients beginning to undergo hemodialysis. Korean J Intern Med 33:970–979. https://doi.org/10.3904/kjim.2016.296
32. Bajaj JS, Tandon P, OʼLeary JG, et al (2018) The Impact of Albumin Use on Resolution of Hyponatremia in Hospitalized Patients With Cirrhosis. Am J Gastroenterol 113:1339. https://doi.org/10.1038/s41395-018-0119-3
33. Bales J, Cho S, Tran TK, et al (2016) The Effect of Hyponatremia and Sodium Variability on Outcomes in Adults with Aneurysmal Subarachnoid Hemorrhage. World Neurosurg 96:340–349. https://doi.org/10.1016/j.wneu.2016.09.005
34. Balling L, Kober L, Schou M, et al (2013) Efficacy and safety of angiotensin-converting enzyme inhibitors in patients with left ventricular systolic dysfunction and hyponatremia. J Card Fail 19:725–730. https://doi.org/10.1016/j.cardfail.2013.09.004
35. Barakat AAE-K, Metwaly AA, Nasr FM, et al (2015) Impact of hyponatremia on frequency of complications in patients with decompensated liver cirrhosis. Electron Physician 7:1349–1358. https://doi.org/10.14661/1349
36. Barber SM, Liebelt BD, Baskin DS (2014) Incidence, Etiology and Outcomes of Hyponatremia after Transsphenoidal Surgery: Experience with 344 Consecutive Patients at a Single Tertiary Center. J Clin Med 3:1199–1219. https://doi.org/10.3390/jcm3041199
37. Basaran S, Yavuz SS, Bali EA, et al (2019) Hyponatremia Is Predictive of HSV-1 Encephalitis among Patients with Viral Encephalitis. Tohoku J Exp Med 247:189–195. https://doi.org/10.1620/tjem.247.189
38. Bashir S, Pervaiz A, Khan HA, et al (2019) Frequency of Hyponatremia in Patients with Hepatic Encephalopathy at a tertiary care hospital. Pak J Med Health Sci 13:306–308
39. Bassi V, Fattoruso O (2020) The Role of Fractional Excretion of Uric Acid in the Differential Diagnosis of Hypotonic Hyponatraemia in Patients with Diuretic Therapy. Cureus 12:e7762. https://doi.org/10.7759/cureus.7762
40. Bavishi C, Ather S, Bambhroliya A, et al (2014) Prognostic significance of hyponatremia among ambulatory patients with heart failure and preserved and reduced ejection fractions. Am J Cardiol 113:1834–1838. https://doi.org/10.1016/j.amjcard.2014.03.017
41. Berardi R, Caramanti M, Castagnani M, et al (2015) Hyponatremia is a predictor of hospital length and cost of stay and outcome in cancer patients. Support Care Cancer 23:3095–3101. https://doi.org/10.1007/s00520-015-2683-z
42. Berardi R, Mocchegiani F, Rinaldi S, et al (2020) Hyponatremia is a Predictor of Clinical Outcome for Resected Biliary Tract Cancers: A Retrospective Single-Center Study. Oncol Ther 8:115–124. https://doi.org/10.1007/s40487-020-00112-6
43. Berardi R, Rinaldi S, Belfiori G, et al (2020) The Role of Hyponatraemia Before Surgery in Patients With Radical Resected Pancreatic Cancer. Clin Med Insights Oncol 14:1179554920936605. https://doi.org/10.1177/1179554920936605
44. Berardi R, Santoni M, Newsom-Davis T, et al (2017) Hyponatremia normalization as an independent prognostic factor in patients with advanced non-small cell lung cancer treated with first-line therapy. Oncotarget 8:23871–23879. https://doi.org/10.18632/oncotarget.13372
45. Berghuis B, van der Palen J, de Haan G-J, et al (2017) Carbamazepine- and oxcarbazepine-induced hyponatremia in people with epilepsy. Epilepsia 58:1227–1233. https://doi.org/10.1111/epi.13777
46. Berndt M, Harbeck B, Lindner U, et al (2015) Hyponatremia Due to Thyrotropin Deficiency: A Fairy Tale? Mayo Clin Proc 90:1305–1307. https://doi.org/10.1016/j.mayocp.2015.05.019
47. Berni A, Malandrino D, Parenti G, et al (2020) Hyponatremia, IL-6, and SARS-CoV-2 (COVID-19) infection: may all fit together? J Endocrinol Invest 43:1137–1139. https://doi.org/10.1007/s40618-020-01301-w
48. Bezinover D, Navabi S, Wang M, et al (2019) Hyponatremia Is Protective Against the Development of Portal Vein Thrombosis in Patients Undergoing Liver Transplant. Transplant Proc 51:1880–1886. https://doi.org/10.1016/j.transproceed.2019.05.014
49. Bhandari SK, Adams AL, Li BH, et al (2020) Sub-acute hyponatraemia more than chronic hyponatraemia is associated with serious falls and hip fractures. Intern Med J 50:1100–1108. https://doi.org/10.1111/imj.14684
50. Bhavnani SP, Kumar A, Coleman CI, et al (2014) The prognostic impact of pre-implantation hyponatremia on morbidity and mortality among patients with left ventricular dysfunction and implantable cardioverter-defibrillators. Europace 16:47–54. https://doi.org/10.1093/europace/eut211
51. Bilello JF, McCullough KA, Dirks RC, et al (2020) Incidence of hyponatremia in patients given levetiracetam vs. phenytoin for early posttraumatic seizure prophylaxis. Am J Surg 220:1503–1505. https://doi.org/10.1016/j.amjsurg.2020.08.042
52. Bilgetekin I, Erturk I, Basal FB, et al (2021) Tolvaptan treatment in hyponatremia due to the syndrome of inappropriate secretion of antidiuretic hormone (SIADH): effects on survival in patients with cancer. Int Urol Nephrol 53:301–307. https://doi.org/10.1007/s11255-020-02623-7
53. Bohl MA, Ahmad S, Jahnke H, et al (2016) Delayed Hyponatremia Is the Most Common Cause of 30-Day Unplanned Readmission After Transsphenoidal Surgery for Pituitary Tumors. Neurosurgery 78:84–90. https://doi.org/10.1227/NEU.0000000000001003
54. Bohl MA, Ahmad S, White WL, Little AS (2018) Implementation of a Postoperative Outpatient Care Pathway for Delayed Hyponatremia Following Transsphenoidal Surgery. Neurosurgery 82:110–117. https://doi.org/10.1093/neuros/nyx151
55. Bokemeyer A, Dziewas R, Wiendl H, et al (2017) Hyponatremia upon presentation to the emergency department - the need for urgent neuroimaging studies. Sci Rep 7:1953. https://doi.org/10.1038/s41598-017-02030-6
56. Bonella BM, Warley F (2017) [Hyponatremia induced by high-dose cyclophosphamide therapy: a retrospective cohort study Cyclophosphamide and Hyponatremia]. Rev Fac Cien Med Univ Nac Cordoba 74:201–206. https://doi.org/10.31053/1853.0605.v74.n3.14766
57. Boursier G, Alméras M, Buthiau D, et al (2015) CT-pro-AVP as a tool for assessment of intravascular volume depletion in severe hyponatremia. Clin Biochem 48:640–645. https://doi.org/10.1016/j.clinbiochem.2015.03.013
58. Boyer S, Gayot C, Bimou C, et al (2019) Prevalence of mild hyponatremia and its association with falls in older adults admitted to an emergency geriatric medicine unit (the MUPA unit). BMC Geriatr 19:265. https://doi.org/10.1186/s12877-019-1282-0
59. Braconnier P, Delforge M, Garjau M, et al (2017) Hyponatremia is a marker of disease severity in HIV-infected patients: a retrospective cohort study. BMC Infect Dis 17:98. https://doi.org/10.1186/s12879-017-2191-5
60. Bravo González-Blas L, García-Gago L, Astudillo-Jarrín D, et al (2020) Analysis of Factors Influencing the Prognostic Significance of Hyponatremia in Peritoneal Dialysis Patients. Am J Nephrol 51:54–64. https://doi.org/10.1159/000504870
61. Bridges E, Altherwi T, Correa JA, Hew-Butler T (2020) Oral Hypertonic Saline Is Effective in Reversing Acute Mild-to-Moderate Symptomatic Exercise-Associated Hyponatremia. Clin J Sport Med 30:8–13. https://doi.org/10.1097/JSM.0000000000000573
62. Brinkkoetter PT, Grundmann F, Ghassabeh PJ, et al (2019) Impact of Resolution of Hyponatremia on Neurocognitive and Motor Performance in Geriatric Patients. Sci Rep 9:12526. https://doi.org/10.1038/s41598-019-49054-8
63. Burke WT, Cote DJ, Iuliano SI, et al (2018) A practical method for prevention of readmission for symptomatic hyponatremia following transsphenoidal surgery. Pituitary 21:25–31. https://doi.org/10.1007/s11102-017-0843-5
64. Burst V, Grundmann F, Kubacki T, et al (2017) Thiazide-Associated Hyponatremia, Report of the Hyponatremia Registry: An Observational Multicenter International Study. Am J Nephrol 45:420–430. https://doi.org/10.1159/000471493
65. Burst V, Grundmann F, Kubacki T, et al (2017) Euvolemic hyponatremia in cancer patients. Report of the Hyponatremia Registry: an observational multicenter international study. Support Care Cancer 25:2275–2283. https://doi.org/10.1007/s00520-017-3638-3
66. Cairns RS, Hew-Butler T (2015) Incidence of Exercise-Associated Hyponatremia and Its Association With Nonosmotic Stimuli of Arginine Vasopressin in the GNW100s Ultra-endurance Marathon. Clin J Sport Med 25:347–354. https://doi.org/10.1097/JSM.0000000000000144
67. Cairns RS, Hew-Butler T (2016) Proof of concept: hypovolemic hyponatremia may precede and augment creatine kinase elevations during an ultramarathon. Eur J Appl Physiol 116:647–655. https://doi.org/10.1007/s00421-015-3324-4
68. Carandang F, Anglemyer A, Longhurst CA, et al (2013) Association between maintenance fluid tonicity and hospital-acquired hyponatremia. J Pediatr 163:1646–1651. https://doi.org/10.1016/j.jpeds.2013.07.020
69. Carcel C, Sato S, Zheng D, et al (2016) Prognostic Significance of Hyponatremia in Acute Intracerebral Hemorrhage: Pooled Analysis of the Intensive Blood Pressure Reduction in Acute Cerebral Hemorrhage Trial Studies. Crit Care Med 44:1388–1394. https://doi.org/10.1097/CCM.0000000000001628
70. Cárdenas A, Solà E, Rodríguez E, et al (2014) Hyponatremia influences the outcome of patients with acute-on-chronic liver failure: an analysis of the CANONIC study. Crit Care 18:700. https://doi.org/10.1186/s13054-014-0700-0
71. Castello LM, Baldrighi M, Panizza A, et al (2017) Efficacy and safety of two different tolvaptan doses in the treatment of hyponatremia in the Emergency Department. Intern Emerg Med 12:993–1001. https://doi.org/10.1007/s11739-016-1508-5
72. Castillo JJ, Glezerman IG, Boklage SH, et al (2016) The occurrence of hyponatremia and its importance as a prognostic factor in a cross-section of cancer patients. BMC Cancer 16:564. https://doi.org/10.1186/s12885-016-2610-9
73. Cavusoglu Y, Kaya H, Eraslan S, Yilmaz MB (2019) Hyponatremia is associated with occurrence of atrial fibrillation in outpatients with heart failure and reduced ejection fraction. Hellenic J Cardiol 60:117–121. https://doi.org/10.1016/j.hjc.2018.03.006
74. Cervellin G, Mitaritonno M, Pedrazzoni M, et al (2014) Prevalence of hyponatremia in femur neck fractures: a one-year survey in an urban emergency department. Adv Orthop 2014:397059. https://doi.org/10.1155/2014/397059
75. Cervellin G, Salvagno G, Bonfanti L, et al (2015) Association of Hyponatremia and Hypovitaminosis D in Ambulatory Adults. J Med Biochem 34:450–454. https://doi.org/10.1515/jomb-2015-0003
76. Chalela R, González-García JG, Chillarón JJ, et al (2016) Impact of hyponatremia on mortality and morbidity in patients with COPD exacerbations. Respir Med 117:237–242. https://doi.org/10.1016/j.rmed.2016.05.003
77. Chan VWQ, Henry MT, Kennedy MP (2020) Hyponatremia and hypercalcemia: a study of a large cohort of patients with lung cancer. Transl Cancer Res 9:222–230. https://doi.org/10.21037/tcr.2019.12.72
78. Chang TI, Kim YL, Kim H, et al (2014) Hyponatremia as a predictor of mortality in peritoneal dialysis patients. PLoS One 9:e111373. https://doi.org/10.1371/journal.pone.0111373
79. Chen K-H, Chen C-Y, Lee C-C, et al (2014) Baseline hyponatremia does not predict two-year mortality in patients with chronic peritoneal dialysis. Ren Fail 36:1371–1375. https://doi.org/10.3109/0886022X.2014.945182
80. Chen S, Zhao J-J, Tong N-W, et al (2014) Randomized, double blinded, placebo-controlled trial to evaluate the efficacy and safety of tolvaptan in Chinese patients with hyponatremia caused by SIADH. J Clin Pharmacol 54:1362–1367. https://doi.org/10.1002/jcph.342
81. Chifu I, Gerstl A, Lengenfelder B, et al (2021) Treatment of symptomatic hyponatremia with hypertonic saline: a real-life observational study. Eur J Endocrinol 184:647–655. https://doi.org/10.1530/EJE-20-1207
82. Chitsazian Z, Zamani B, Mohagheghfar M (2013) Prevalence of hyponatremia in intensive care unit patients with brain injury in kashan shahid-beheshti hospital in 2012. Arch Trauma Res 2:91–94. https://doi.org/10.5812/atr.9877
83. Chlíbková D, Knechtle B, Rosemann T, et al (2015) Rhabdomyolysis and exercise-associated hyponatremia in ultra-bikers and ultra-runners. J Int Soc Sports Nutr 12:29. https://doi.org/10.1186/s12970-015-0091-x
84. Chlíbková D, Knechtle B, Rosemann T, et al (2014) The prevalence of exercise-associated hyponatremia in 24-hour ultra-mountain bikers, 24-hour ultra-runners and multi-stage ultra-mountain bikers in the Czech Republic. J Int Soc Sports Nutr 11:3. https://doi.org/10.1186/1550-2783-11-3
85. Choi EY, Park J-S, Kim YT, et al (2015) The risk of hyponatremia with desmopressin use for nocturnal polyuria. Am J Nephrol 41:183–190. https://doi.org/10.1159/000381562
86. Choi EY, Ro Y, Choi J-W, et al (2016) Cicletanine-induced hyponatremia and hypokalemia in kidney transplant patients. Kidney Res Clin Pract 35:142–146. https://doi.org/10.1016/j.krcp.2016.05.004
87. Choi JS, Kim CS, Bae EH, et al (2017) Prognostic impact of hyponatremia occurring at various time points during hospitalization on mortality in patients with acute myocardial infarction. Medicine (Baltimore) 96:e7023. https://doi.org/10.1097/MD.0000000000007023
88. Chung M-C, Yu T-M, Shu K-H, et al (2017) Hyponatremia and increased risk of dementia: A population-based retrospective cohort study. PLoS One 12:e0178977. https://doi.org/10.1371/journal.pone.0178977
89. Correia L, Ferreira R, Correia I, et al (2014) Severe hyponatremia in older patients at admission in an internal medicine department. Arch Gerontol Geriatr 59:642–647. https://doi.org/10.1016/j.archger.2014.08.002
90. Crestanello JA, Phillips G, Firstenberg MS, et al (2013) Postoperative hyponatremia predicts an increase in mortality and in-hospital complications after cardiac surgery. J Am Coll Surg 216:1135–1143, 1143.e1. https://doi.org/10.1016/j.jamcollsurg.2013.02.010
91. Crestanello JA, Phillips G, Firstenberg MS, et al (2013) Does preoperative hyponatremia potentiate the effects of left ventricular dysfunction on mortality after cardiac surgery? J Thorac Cardiovasc Surg 145:1589–1594, 1594.e1–2. https://doi.org/10.1016/j.jtcvs.2012.12.093
92. Crestanello JA, Phillips G, Firstenberg MS, et al (2013) Preoperative hyponatremia predicts outcomes after cardiac surgery. J Surg Res 181:60–66. https://doi.org/10.1016/j.jss.2012.06.004
93. Cumming K, Hoyle GE, Hutchison JD, Soiza RL (2014) Prevalence, incidence and etiology of hyponatremia in elderly patients with fragility fractures. PLoS One 9:e88272. https://doi.org/10.1371/journal.pone.0088272
94. Cumming K, McKenzie S, Hoyle GE, et al (2015) Prognosis of hyponatremia in elderly patients with fragility fractures. J Clin Med Res 7:45–51. https://doi.org/10.14740/jocmr1984w
95. Czupryna P, Moniuszko A, Garkowski A, et al (2016) Comparison of hyponatremia and SIADH frequency in patients with tick borne encephalitis and meningitis of other origin. Scand J Clin Lab Invest 76:159–164. https://doi.org/10.3109/00365513.2015.1129669
96. Daher EDF, Soares D de S, Filho SLAP, et al (2017) Hyponatremia and risk factors for death in human visceral leishmaniasis: new insights from a cross-sectional study in Brazil. BMC Infect Dis 17:168. https://doi.org/10.1186/s12879-017-2257-4
97. Danz M, Pöttgen K, Tönjes PM, et al (2016) Hyponatremia among Triathletes in the Ironman European Championship. N Engl J Med 374:997–998. https://doi.org/10.1056/NEJMc1510409
98. Dash SC, Sundaray NK, Rajesh B, Pagad T (2019) Hyponatremia in Elderly In-Patients. J Clin Diagn Res 13:. https://doi.org/10.7860/JCDR/2019/39957.12554
99. Dasta J, Waikar SS, Xie L, et al (2015) Patterns of treatment and correction of hyponatremia in intensive care unit patients. J Crit Care 30:1072–1079. https://doi.org/10.1016/j.jcrc.2015.06.016
100. Dayrit JCP, Cunanan EC, Kho SA (2016) Prevalence of Hyponatremia in Hypothyroid Patients during Radioactive 131I Ablation for Differentiated Thyroid Cancer: Single Institution Experience. Endocrinol Metab (Seoul) 31:410–415. https://doi.org/10.3803/EnM.2016.31.3.410
101. De Vecchis R, Di Maio M, Di Biase G, Ariano C (2016) Effects of Hyponatremia Normalization on the Short-Term Mortality and Rehospitalizations in Patients with Recent Acute Decompensated Heart Failure: A Retrospective Study. J Clin Med 5:E92. https://doi.org/10.3390/jcm5100092
102. Deaver KE, Catel CP, Lillehei KO, et al (2018) Strategies to reduce readmissions for hyponatremia after transsphenoidal surgery for pituitary adenomas. Endocrine 62:333–339. https://doi.org/10.1007/s12020-018-1656-7
103. Decaux G, Musch W (2019) Estimated Daily Urine Volume and Solute Excretion from Spot Urine Samples to Guide the Therapy of Hyponatremia in SIADH. J Clin Med 8:E1511. https://doi.org/10.3390/jcm8101511
104. Deitelzweig S, Amin A, Christian R, et al (2013) Health care utilization, costs, and readmission rates associated with hyponatremia. Hosp Pract (1995) 41:89–95. https://doi.org/10.3810/hp.2013.02.1014
105. Deitelzweig S, Amin A, Christian R, et al (2013) Hyponatremia-associated healthcare burden among US patients hospitalized for cirrhosis. Adv Ther 30:71–80. https://doi.org/10.1007/s12325-012-0073-1
106. Dekker MJE, Marcelli D, Canaud B, et al (2016) Unraveling the relationship between mortality, hyponatremia, inflammation and malnutrition in hemodialysis patients: results from the international MONDO initiative. Eur J Clin Nutr 70:779–784. https://doi.org/10.1038/ejcn.2016.49
107. Demir TA, Yılmaz F, Sönmez BM, et al (2019) Association of optic nerve sheath diameter measurement with hyponatremia in emergency department. Am J Emerg Med 37:1876–1879. https://doi.org/10.1016/j.ajem.2018.12.054
108. Der-Nigoghossian C, Lesch C, Berger K (2017) Effectiveness and Tolerability of Conivaptan and Tolvaptan for the Treatment of Hyponatremia in Neurocritically Ill Patients. Pharmacotherapy 37:528–534. https://doi.org/10.1002/phar.1926
109. Dhrolia MF, Akhtar SF, Ahmed E, et al (2014) Azotemia protects the brain from osmotic demyelination on rapid correction of hyponatremia. Saudi J Kidney Dis Transpl 25:558–566. https://doi.org/10.4103/1319-2442.132183
110. Diaconu CC, Manea M, Iancu MA, et al (2018) Hyponatremia in Patients with Heart Failure: a Prognostic Marker. Rev Chim 69:1071–1074
111. Diemar SS, Sejling A-S, Eiken P, et al (2019) Hyponatremia and metabolic bone disease in patients with epilepsy: A cross-sectional study. Bone 123:67–75. https://doi.org/10.1016/j.bone.2019.03.017
112. Diker-Cohen T, Rozen-Zvi B, Yelin D, et al (2018) Endocrinopathy-induced euvolemic hyponatremia. Intern Emerg Med 13:679–688. https://doi.org/10.1007/s11739-018-1872-4
113. Dimitriadis C, Sekercioglu N, Pipili C, et al (2014) Hyponatremia in peritoneal dialysis: epidemiology in a single center and correlation with clinical and biochemical parameters. Perit Dial Int 34:260–270. https://doi.org/10.3747/pdi.2012.00095
114. Dominguez M, Perez JA, Patel CB (2013) Efficacy of 3% saline vs. conivaptan in achieving hyponatremia treatment goals. Methodist Debakey Cardiovasc J 9:49–53. https://doi.org/10.14797/mdcj-9-1-49
115. Donzé JD, Beeler PE, Bates DW (2016) Impact of Hyponatremia Correction on the Risk for 30-Day Readmission and Death in Patients with Congestive Heart Failure. Am J Med 129:836–842. https://doi.org/10.1016/j.amjmed.2016.02.036
116. Dunlap ME, Hauptman PJ, Amin AN, et al (2017) Current Management of Hyponatremia in Acute Heart Failure: A Report From the Hyponatremia Registry for Patients With Euvolemic and Hypervolemic Hyponatremia (HN Registry). J Am Heart Assoc 6:e005261. https://doi.org/10.1161/JAHA.116.005261
117. Eckart A, Hausfater P, Amin D, et al (2018) Hyponatremia and activation of vasopressin secretion are both independently associated with 30-day mortality: results of a multicenter, observational study. J Intern Med 284:270–281. https://doi.org/10.1111/joim.12764
118. Ehtesham M, Mohmand M, Raj K, et al (2019) Clinical Spectrum of Hyponatremia in Patients with Stroke. Cureus 11:e5310. https://doi.org/10.7759/cureus.5310
119. Elmi G, Zaccaroni S, Arienti V, Faustini-Fustini M (2014) Prevalence and in-hospital mortality of hyponatremia: a cohort study. Eur J Intern Med 25:e45-46. https://doi.org/10.1016/j.ejim.2014.03.013
120. Ennaifer R, Cheikh M, Romdhane H, et al (2016) Hyponatremia in cirrhosis: Risk factors and prognostic value. Tunis Med 94:401–405
121. Escamilla-Ocañas CE, Venkatasubba Rao CP, Bershad E, Damani R (2020) Temporal Relationship between Hyponatremia and Development of Cerebral Vasospasm in Aneurysmal Subarachnoid Hemorrhage Patients: A Retrospective Observational Study. J Stroke Cerebrovasc Dis 29:104789. https://doi.org/10.1016/j.jstrokecerebrovasdis.2020.104789
122. Ezoe Y, Mizusawa J, Katayama H, et al (2018) An integrated analysis of hyponatremia in cancer patients receiving platinum-based or nonplatinum-based chemotherapy in clinical trials (JCOG1405-A). Oncotarget 9:6595–6606. https://doi.org/10.18632/oncotarget.23536
123. Fahlén Bergh C, Toivanen S, Johnell K, et al (2020) Factors of importance for discontinuation of thiazides associated with hyponatremia in Sweden: A population-based register study. Pharmacoepidemiol Drug Saf 29:77–83. https://doi.org/10.1002/pds.4922
124. Falhammar H, Calissendorff J, Skov J, et al (2019) Tramadol- and codeine-induced severe hyponatremia: A Swedish population-based case-control study. Eur J Intern Med 69:20–24. https://doi.org/10.1016/j.ejim.2019.08.006
125. Falhammar H, Lindh JD, Calissendorff J, et al (2018) Differences in associations of antiepileptic drugs and hospitalization due to hyponatremia: A population-based case-control study. Seizure 59:28–33. https://doi.org/10.1016/j.seizure.2018.04.025
126. Falhammar H, Lindh JD, Calissendorff J, et al (2019) Antipsychotics and severe hyponatremia: A Swedish population-based case-control study. Eur J Intern Med 60:71–77. https://doi.org/10.1016/j.ejim.2018.11.011
127. Falhammar H, Lindh JD, Calissendorff J, et al (2019) Associations of proton pump inhibitors and hospitalization due to hyponatremia: A population-based case-control study. Eur J Intern Med 59:65–69. https://doi.org/10.1016/j.ejim.2018.08.012
128. Falhammar H, Skov J, Calissendorff J, et al (2020) Inverse association between glucose-lowering medications and severe hyponatremia: a Swedish population-based case-control study. Endocrine 67:579–586. https://doi.org/10.1007/s12020-019-02160-z
129. Falhammar H, Skov J, Calissendorff J, et al (2020) Associations Between Antihypertensive Medications and Severe Hyponatremia: A Swedish Population-Based Case-Control Study. J Clin Endocrinol Metab 105:dgaa194. https://doi.org/10.1210/clinem/dgaa194
130. Fan S-S, Lin L-F, Chen VC-H, et al (2020) Effects of Lower Past-Year Serum Sodium and Hyponatremia on Depression Symptoms and Cognitive Impairments in Patients With Hemodialysis. Ther Apher Dial 24:169–177. https://doi.org/10.1111/1744-9987.13395
131. Farmand S, Lindh JD, Calissendorff J, et al (2018) Differences in Associations of Antidepressants and Hospitalization Due to Hyponatremia. Am J Med 131:56–63. https://doi.org/10.1016/j.amjmed.2017.07.025
132. Fawad A, Hussain A, Hussain SL, et al (2020) Examine the Frequency and Short Term Outcomes of Hyponatremia in Patients with Acute ST Elevation Myocardial Infarction. Pak J Med Health Sci 14:651–653
133. Fehlberg EA, Lucero RJ, Weaver MT, et al (2017) Associations between hyponatraemia, volume depletion and the risk of falls in US hospitalised patients: A case-control study. BMJ Open 7:. https://doi.org/10.1136/bmjopen-2017-017045
134. Feinstein AJ, Davis J, Gonzalez L, et al (2016) Hyponatremia and perioperative complications in patients with head and neck squamous cell carcinoma. Head Neck 38 Suppl 1:E1370-1374. https://doi.org/10.1002/hed.24229
135. Feinstein Y, Yerushalmi B, Loewenthal N, et al (2014) Natural history and clinical manifestations of hyponatremia and hyperchlorhidrosis due to carbonic anhydrase XII deficiency. Horm Res Paediatr 81:336–342. https://doi.org/10.1159/000358327
136. Filmyer DM, Finkel G, Plasay M, et al (2019) A comparison of simple reaction times in psychotic inpatients with and without hyponatremia. Schizophr Res 208:449–450. https://doi.org/10.1016/j.schres.2019.04.001
137. Formiga F, Chivite D, Brasé A, et al (2018) Clinical characteristics and prognosis in patients with a first acute heart failure hospitalization according to admission hyponatremia. Acta Clin Belg 73:281–286. https://doi.org/10.1080/17843286.2018.1429345
138. Fournier J-P, Yin H, Nessim SJ, et al (2015) Tramadol for noncancer pain and the risk of hyponatremia. Am J Med 128:418-425.e5. https://doi.org/10.1016/j.amjmed.2014.10.046
139. Fralick M, Schneeweiss S, Wallis CJD, et al (2019) Desmopressin and the risk of hyponatremia: A population-based cohort study. PLoS Med 16:e1002930. https://doi.org/10.1371/journal.pmed.1002930
140. Frontera JA, Valdes E, Huang J, et al (2020) Prevalence and Impact of Hyponatremia in Patients With Coronavirus Disease 2019 in New York City. Crit Care Med 48:e1211–e1217. https://doi.org/10.1097/CCM.0000000000004605
141. Fucà G, Mariani L, Lo Vullo S, et al (2019) Weighing the prognostic role of hyponatremia in hospitalized patients with metastatic solid tumors: the HYPNOSIS study. Sci Rep 9:12993. https://doi.org/10.1038/s41598-019-49601-3
142. Fujisawa C, Umegaki H, Sugimoto T, et al (2021) Mild hyponatremia is associated with low skeletal muscle mass, physical function impairment, and depressive mood in the elderly. BMC Geriatr 21:15. https://doi.org/10.1186/s12877-020-01955-4
143. Furukawa J, Miyake H, Kusuda Y, Fujisawa M (2015) Hyponatremia as a powerful prognostic predictor for Japanese patients with clear cell renal cell carcinoma treated with a tyrosine kinase inhibitor. Int J Clin Oncol 20:351–357. https://doi.org/10.1007/s10147-014-0713-3
144. Gala-Błądzińska A, Czarnota J, Kaczorowski R, et al (2019) Mild hyponatremia discovered within the first 24 hours of ischemic stroke is a risk factor for early post stroke mortality. Adv Clin Exp Med 28:1321–1327. https://doi.org/10.17219/acem/103070
145. Galluzzo A, Frea S, Boretto P, et al (2020) Spot urinary sodium in acute decompensation of advanced heart failure and dilutional hyponatremia: insights from DRAIN trial. Clin Res Cardiol 109:1251–1259. https://doi.org/10.1007/s00392-020-01617-w
146. Gandhi S, McArthur E, Mamdani MM, et al (2016) Antiepileptic drugs and hyponatremia in older adults: Two population-based cohort studies. Epilepsia 57:2067–2079. https://doi.org/10.1111/epi.13593
147. Gandhi S, McArthur E, Reiss JP, et al (2016) Atypical antipsychotic medications and hyponatremia in older adults: a population-based cohort study. Can J Kidney Health Dis 3:21. https://doi.org/10.1186/s40697-016-0111-z
148. Gandhi S, Shariff SZ, Al-Jaishi A, et al (2017) Second-Generation Antidepressants and Hyponatremia Risk: A Population-Based Cohort Study of Older Adults. Am J Kidney Dis 69:87–96. https://doi.org/10.1053/j.ajkd.2016.08.020
149. Gang X, Zhang Y, Pan X, et al (2018) Hyponatremia: Prevalence and characteristics in internal medicine patients in southeast of China. Medicine (Baltimore) 97:e13389. https://doi.org/10.1097/MD.0000000000013389
150. Ganguli A, Mascarenhas RC, Jamshed N, et al (2015) Hyponatremia: incidence, risk factors, and consequences in the elderly in a home-based primary care program. Clin Nephrol 84:75–85. https://doi.org/10.5414/CN108453
151. Gankam-Kengne F, Ayers C, Khera A, et al (2013) Mild hyponatremia is associated with an increased risk of death in an ambulatory setting. Kidney Int 83:700–706. https://doi.org/10.1038/ki.2012.459
152. García-Sanz M-T, Martínez-Gestoso S, Calvo-Álvarez U, et al (2020) Impact of Hyponatremia on COPD Exacerbation Prognosis. J Clin Med 9:E503. https://doi.org/10.3390/jcm9020503
153. Garrahy A, Dineen R, Hannon AM, et al (2019) Continuous Versus Bolus Infusion of Hypertonic Saline in the Treatment of Symptomatic Hyponatremia Caused by SIAD. J Clin Endocrinol Metab 104:3595–3602. https://doi.org/10.1210/jc.2019-00044
154. Gasparotto APDC, Falcão ALE, Kosour C, et al (2016) Atrial natriuretic factor: is it responsible for hyponatremia and natriuresis in neurosurgery? Rev Bras Ter Intensiva 28:154–160. https://doi.org/10.5935/0103-507X.20160030
155. Gefen S, Joffe E, Mayan H, Justo D (2014) Recurrent hospitalizations with moderate to severe hyponatremia in older adults and its associated mortality. Eur J Intern Med 25:624–628. https://doi.org/10.1016/j.ejim.2014.06.020
156. Geoghegan P, Harrison AM, Thongprayoon C, et al (2015) Sodium Correction Practice and Clinical Outcomes in Profound Hyponatremia. Mayo Clin Proc 90:1348–1355. https://doi.org/10.1016/j.mayocp.2015.07.014
157. George JC, Zafar W, Bucaloiu ID, Chang AR (2018) Risk Factors and Outcomes of Rapid Correction of Severe Hyponatremia. Clin J Am Soc Nephrol 13:984–992. https://doi.org/10.2215/CJN.13061117
158. Gilboa M, Koren G, Katz R, et al (2019) Anxiolytic treatment but not anxiety itself causes hyponatremia among anxious patients. Medicine (Baltimore) 98:e14334. https://doi.org/10.1097/MD.0000000000014334
159. Giordano M, Ciarambino T, Castellino P, et al (2017) Seasonal variations of hyponatremia in the emergency department: Age-related changes. Am J Emerg Med 35:749–752. https://doi.org/10.1016/j.ajem.2017.01.018
160. Giordano M, Ciarambino T, Priore EL, et al (2017) Serum sodium correction rate and the outcome in severe hyponatremia. Am J Emerg Med 35:1691–1694. https://doi.org/10.1016/j.ajem.2017.05.050
161. Golestaneh L, Neugarten J, Kaskel F, McGinn AP (2018) Progressive kidney disease may not alter the association of hyponatremia with mortality. Clin Exp Nephrol 22:889–897. https://doi.org/10.1007/s10157-018-1536-8
162. Golestaneh L, Neugarten J, Southern W, et al (2017) Improving the diagnostic workup of hyponatremia in the setting of kidney disease: a continuing medical education (CME) initiative. Int Urol Nephrol 49:491–497. https://doi.org/10.1007/s11255-017-1501-6
163. Gómez-Hoyos E, Buigues AO, Ballesteros Pomar MD, et al (2019) Development of hyponatremia in non-critical patients receiving total parenteral nutrition: A prospective, multicenter study. Clin Nutr 38:2639–2644. https://doi.org/10.1016/j.clnu.2018.11.014
164. Gómez-Hoyos E, Cuesta M, Del Prado-González N, et al (2017) Prevalence of Hyponatremia and Its Associated Morbimortality in Hospitalized Patients Receiving Parenteral Nutrition. Ann Nutr Metab 71:1–7. https://doi.org/10.1159/000477675
165. Gómez-Hoyos E, Fernández-Peña S, Cuesta M, et al (2018) Hyponatremia in patients receiving parenteral nutrition: the importance of correcting serum sodium for total proteins. The role of the composition of parenteral nutrition in the development of hyponatremia. Eur J Clin Nutr 72:446–451. https://doi.org/10.1038/s41430-017-0026-5
166. Goren I, Israel A, Carmel-Neiderman NN, et al (2016) Vomiting and Hyponatremia Are Risk Factors for Worse Clinical Outcomes Among Patients Hospitalized Due to Nonsurgical Abdominal Pain: A Retrospective Cohort Study. Medicine (Baltimore) 95:e3274. https://doi.org/10.1097/MD.0000000000003274
167. Gota V, Kavathiya K, Doshi K, et al (2014) High plasma exposure to pemetrexed leads to severe hyponatremia in patients with advanced non small cell lung cancer receiving pemetrexed-platinum doublet chemotherapy. Cancer Manag Res 6:261–265. https://doi.org/10.2147/CMAR.S60177
168. Goyal A, Mezue K, Rangaswami J (2017) Visit-to-visit systolic blood pressure variability predicts treatment-related adverse event of hyponatremia in SPRINT. Cardiovasc Ther 35:. https://doi.org/10.1111/1755-5922.12274
169. Gralla RJ, Ahmad F, Blais JD, et al (2017) Tolvaptan use in cancer patients with hyponatremia due to the syndrome of inappropriate antidiuretic hormone: a post hoc analysis of the SALT-1 and SALT-2 trials. Cancer Med 6:723–729. https://doi.org/10.1002/cam4.805
170. Grattagliano I, Mastronuzzi T, D’Ambrosio G (2018) Hyponatremia associated with long-term medication use in the elderly: an analysis in general practice. J Prim Health Care 10:167–173. https://doi.org/10.1071/HC17084
171. Gray JR, Morbitzer KA, Liu-DeRyke X, et al (2014) Hyponatremia in Patients with Spontaneous Intracerebral Hemorrhage. J Clin Med 3:1322–1332. https://doi.org/10.3390/jcm3041322
172. Greenberg A, Verbalis JG, Amin AN, et al (2015) Current treatment practice and outcomes. Report of the hyponatremia registry. Kidney Int 88:167–177. https://doi.org/10.1038/ki.2015.4
173. Gritti P, Lanterna LA, Rotasperti L, et al (2014) Clinical evaluation of hyponatremia and hypovolemia in critically ill adult neurologic patients: contribution of the use of cumulative balance of sodium. J Anesth 28:687–695. https://doi.org/10.1007/s00540-014-1814-x
174. Gu A, Chen FR, Chen AZ, et al (2020) Preoperative hyponatremia is an independent risk factor for postoperative complications in aseptic revision hip and knee arthroplasty. J Orthop 20:224–227. https://doi.org/10.1016/j.jor.2020.01.028
175. Gunathilake R, Oldmeadow C, McEvoy M, et al (2013) Mild hyponatremia is associated with impaired cognition and falls in community-dwelling older persons. J Am Geriatr Soc 61:1838–1839. https://doi.org/10.1111/jgs.12468
176. Guo J-Y, Gong T-T, Yang Z, et al (2019) Prognostic value of preoperative hyponatremia in patients with epithelial ovarian cancer. J Cancer 10:836–842. https://doi.org/10.7150/jca.28118
177. Gupta DK, Bhoi SK, Kalita J, Misra UK (2015) Hyponatremia following esclicarbazepine therapy. Seizure 29:11–14. https://doi.org/10.1016/j.seizure.2015.03.005
178. Gupta S, Tio MC, Gutowski ED, et al (2020) Incidence of Hyponatremia in Patients With Indwelling Peritoneal Catheters for Drainage of Malignant Ascites. JAMA Netw Open 3:e2017859. https://doi.org/10.1001/jamanetworkopen.2020.17859
179. Hagino T, Ochiai S, Watanabe Y, et al (2013) Hyponatremia at admission is associated with in-hospital death in patients with hip fracture. Arch Orthop Trauma Surg 133:507–511. https://doi.org/10.1007/s00402-013-1693-x
180. Hamaguchi S, Kinugawa S, Tsuchihashi-Makaya M, et al (2014) Hyponatremia is an independent predictor of adverse clinical outcomes in hospitalized patients due to worsening heart failure. J Cardiol 63:182–188. https://doi.org/10.1016/j.jjcc.2013.07.012
181. Hammami MM, Almogbel F, Hammami S, et al (2013) Acute severe hypothyroidism is not associated with hyponatremia even with increased water intake: a prospective study in thyroid cancer patients. BMC Endocr Disord 13:27. https://doi.org/10.1186/1472-6823-13-27
182. Han SS, Han M, Park JY, et al (2016) Posttransplant Hyponatremia Predicts Graft Failure and Mortality in Kidney Transplantation Recipients: A Multicenter Cohort Study in Korea. PLoS One 11:e0156050. https://doi.org/10.1371/journal.pone.0156050
183. Han SW, Yi JH, Kang KP, et al (2018) Safety and Efficacy of Tolvaptan in Korean Patients with Hyponatremia Caused by the Syndrome of Inappropriate Antidiuretic Hormone. J Korean Med Sci 33:e112. https://doi.org/10.3346/jkms.2018.33.e112
184. Han X, Li J, Yang J-M, et al (2020) A retrospective analysis of hyponatremia during terlipressin treatment in patients with esophageal or gastric variceal bleeding due to portal hypertension. JGH Open 4:368–370. https://doi.org/10.1002/jgh3.12254
185. Hannon MJ, Behan LA, O’Brien MMC, et al (2014) Hyponatremia following mild/moderate subarachnoid hemorrhage is due to SIAD and glucocorticoid deficiency and not cerebral salt wasting. J Clin Endocrinol Metab 99:291–298. https://doi.org/10.1210/jc.2013-3032
186. Hao J, Li Y, Zhang X, et al (2017) The prevalence and mortality of hyponatremia is seriously underestimated in Chinese general medical patients: an observational retrospective study. BMC Nephrol 18:328. https://doi.org/10.1186/s12882-017-0744-x
187. Harbeck B, Lindner U, Haas CS (2016) Low-dose tolvaptan for the treatment of hyponatremia in the syndrome of inappropriate ADH secretion (SIADH). Endocrine 53:872–873. https://doi.org/10.1007/s12020-016-0912-y
188. Hatakeyama S, Shida T, Yamaguchi H (2019) Risk Factors for Severe Hyponatremia Related to Cisplatin: A Retrospective Case-Control Study. Biol Pharm Bull 42:1891–1897. https://doi.org/10.1248/bpb.b19-00477
189. Hauptman PJ, Burnett J, Gheorghiade M, et al (2013) Clinical course of patients with hyponatremia and decompensated systolic heart failure and the effect of vasopressin receptor antagonism with tolvaptan. J Card Fail 19:390–397. https://doi.org/10.1016/j.cardfail.2013.04.001
190. Hausman-Kedem M, Reif S, Danino D, et al (2018) Mechanism of Hyponatremia in Community-Acquired Pneumonia: Does B-type Natriuretic Peptide Play a Causative Role? Pediatr Emerg Care 34:641–646. https://doi.org/10.1097/PEC.0000000000000814
191. Hayashi M, Abe K, Fujita M, et al (2018) Association between the Serum Sodium Levels and the Response to Tolvaptan in Liver Cirrhosis Patients with Ascites and Hyponatremia. Intern Med 57:2451–2458. https://doi.org/10.2169/internalmedicine.0629-17
192. Hefler-Frischmuth K, Grimm C, Gensthaler L, et al (2018) Prognostic value of preoperative hyponatremia and thrombocytosis in patients with epithelial ovarian cancer. Wien Klin Wochenschr 130:575–580. https://doi.org/10.1007/s00508-018-1388-y
193. Hellman T, Uusalo P, Järvisalo MJ (2021) Continuous hemodialysis with citrate anticoagulation and standard dialysate for managing acute kidney injury in patients with moderate to severe hyponatremia-A retrospective study. Acta Anaesthesiol Scand 65:778–784. https://doi.org/10.1111/aas.13798
194. Hennrikus E, Ou G, Kinney B, et al (2015) Prevalence, Timing, Causes, and Outcomes of Hyponatremia in Hospitalized Orthopaedic Surgery Patients. J Bone Joint Surg Am 97:1824–1832. https://doi.org/10.2106/JBJS.O.00103
195. Hoffman H, Ziechmann R, Gould G, Chin LS (2018) The Impact of Aneurysm Location on Incidence and Etiology of Hyponatremia Following Subarachnoid Hemorrhage. World Neurosurg 110:e621–e626. https://doi.org/10.1016/j.wneu.2017.11.058
196. Hoffman MD, Fogard K, Winger J, et al (2013) Characteristics of 161-km ultramarathon finishers developing exercise-associated hyponatremia. Res Sports Med 21:164–175. https://doi.org/10.1080/15438627.2012.757230
197. Hoffman MD, Hew-Butler T, Stuempfle KJ (2013) Exercise-associated hyponatremia and hydration status in 161-km ultramarathoners. Med Sci Sports Exerc 45:784–791. https://doi.org/10.1249/MSS.0b013e31827985a8
198. Hoffman MD, Stuempfle KJ, Valentino T (2015) Sodium Intake During an Ultramarathon Does Not Prevent Muscle Cramping, Dehydration, Hyponatremia, or Nausea. Sports Med Open 1:39. https://doi.org/10.1186/s40798-015-0040-x
199. Hoffman MD, Stuempfle KJ (2015) Sodium Supplementation and Exercise-Associated Hyponatremia during Prolonged Exercise. Med Sci Sports Exerc 47:1781–1787. https://doi.org/10.1249/MSS.0000000000000599
200. Hoffman MD (2019) Predicted Risk for Exacerbation of Exercise-Associated Hyponatremia from Indiscriminate Postrace Intravenous Hydration of Ultramarathon Runners. J Emerg Med 56:177–184. https://doi.org/10.1016/j.jemermed.2018.10.026
201. Hoffmann DB, Popescu C, Komrakova M, Welte L, Saul D, Lehmann W, Hawellek T, Beil FT, Dakna M, Sehmisch S (2019) Chronic hyponatremia in patients with proximal femoral fractures after low energy trauma: A retrospective study in a level-1 trauma center. Bone Rep 12:100234. https://doi.org/10.1016/j.bonr.2019.100234
202. Holland-Bill L, Christiansen CF, Farkas DK, Donskov F, Jørgensen JOL, Sørensen HT (2018) Diagnosis of hyponatremia and increased risk of a subsequent cancer diagnosis: results from a nationwide population-based cohort study. Acta Oncol 57:522-527. https://doi.org/10.1080/0284186X.2017.1378430
203. Holland-Bill L, Christiansen CF, Heide-Jørgensen U, Ulrichsen SP, Ring T, Jørgensen JO, Sørensen HT (2015) Hyponatremia and mortality risk: a Danish cohort study of 279 508 acutely hospitalized patients. Eur J Endocrinol 173:71-81. https://doi.org/10.1530/EJE-15-0111
204. Holland-Bill L, Christiansen CF, Ulrichsen SP, Ring T, Lunde Jørgensen JO, Sørensen HAT (2019) Preadmission Diuretic Use and Mortality in Patients Hospitalized With Hyponatremia: A Propensity Score-Matched Cohort Study. Am J Ther 26:e79-e91. https://doi.org/10.1097/MJT.0000000000000544
205. Holm JP, Amar AOS, Hyldstrup L, Jensen JEB (2016) Hyponatremia, a risk factor for osteoporosis and fractures in women. Osteoporos Int 27:989-1001. https://doi.org/10.1007/s00198-015-3370-0
206. Holmes BB, Patel N, Lugo R, Richardson T, Metawee M, Rinke LL, Meisch C, Shoemaker MB, Ellis CR (2019) Clinical predictors of acute hyponatremia following LARIAT ligation of the left atrial appendage. J Cardiovasc Electrophysiol 30:2501-2507. https://doi.org/10.1111/jce.14177
207. Hosseini SR, Baghitabar N, Mirzapour A, Oliaei F, Nooreddini H, Bijani A, Mouodi S (2018) Hyponatremia, bone mineral density and falls in the elderly; Results from AHAP study. Rom J Intern Med 56:41-46. https://doi.org/10.1515/rjim-2017-0032
208. Huang CC, Chung CM, Hung SI, Pan WH, Leu HB, Huang PH, Chiu CC, Lin LY, Lin CC, Yang CY, Li SY, Chen YC, Wu TC, Lin SJ, Chen JW (2015) Clinical and Genetic Factors Associated With Thiazide-Induced Hyponatremia. Medicine (Baltimore) 94:e1422. https://doi.org/10.1097/MD.0000000000001422
209. Human T, Cook AM, Anger B, Bledsoe K, Castle A, Deen D, Gibbs H, Lesch C, Liang N, McAllen K, Morrison C, Parker D Jr, Rowe AS, Rhoney D, Sangha K, Santayana E, Taylor S, Tesoro E, Brophy G (2017) Treatment of Hyponatremia in Patients with Acute Neurological Injury. Neurocrit Care 27:242-248. https://doi.org/10.1007/s12028-016-0343-x
210. Hussain NS, Piper M, Ludlam WG, Ludlam WH, Fuller CJ, Mayberg MR (2013) Delayed postoperative hyponatremia after transsphenoidal surgery: prevalence and associated factors. J Neurosurg 119:1453-60. https://doi.org/10.3171/2013.8.JNS13411
211. Ideguchi T, Tsuruda T, Sato Y, Kitamura K (2016) Coexisting Hyponatremia and Decline in Diastolic Blood Pressure Predispose to Atrial Standstill in Hyperkalemic Patients. Circ J 80:1781-6. https://doi.org/10.1253/circj.CJ-16-0283
212. Il Shin J, Park SJ, Suh CH, Lee GH, Hur MW, Han SY, Kim DS, Kim JH (2016) Hyponatremia in patients with systemic lupus erythematosus. Sci Rep 6:25566. https://doi.org/10.1038/srep25566
213. Imai N, Osako K, Kaneshiro N, Shibagaki Y (2018) Seasonal prevalence of hyponatremia in the emergency department: impact of age. BMC Emerg Med 18:41. https://doi.org/10.1186/s12873-018-0182-5
214. Imamura T, Kinugawa K, Minatsuki S, Muraoka H, Kato N, Inaba T, Maki H, Hatano M, Yao A, Komuro I (2014) Urine sodium excretion after tolvaptan administration is dependent upon baseline serum sodium levels: a possible explanation for the improvement of hyponatremia with scarce chance of hypernatremia by a vasopressin receptor antagonist. Int Heart J 55:131-7. https://doi.org/10.1536/ihj.13-221
215. Imamura T, Kinugawa K (2016) Prognostic Impacts of Hyponatremia, Renal Dysfunction, and High-Dose Diuretics During a 10-Year Study Period in 4,087 Japanese Heart Failure Patients. Int Heart J 57:657-8. https://doi.org/10.1536/ihj.16-227
216. Isemann B, Mueller EW, Narendran V, Akinbi H (2016) Impact of Early Sodium Supplementation on Hyponatremia and Growth in Premature Infants: A Randomized Controlled Trial. JPEN J Parenter Enteral Nutr 40:342-9. https://doi.org/10.1177/0148607114558303
217. Israel A, Grossman E (2017) Elevated High-Density Lipoprotein Cholesterol Is Associated with Hyponatremia in Hypertensive Patients. Am J Med 130:1324.e7-1324.e13. https://doi.org/10.1016/j.amjmed.2017.05.030
218. Jahangiri A, Wagner J, Tran MT, Miller LM, Tom MW, Kunwar S, Blevins L Jr, Aghi MK (2013) Factors predicting postoperative hyponatremia and efficacy of hyponatremia management strategies after more than 1000 pituitary operations. J Neurosurg 119:1478-83. https://doi.org/10.3171/2013.7.JNS13273
219. Jain AK, Nandy P (2019) Clinico-etiological profile of hyponatremia among elderly age group patients in a tertiary care hospital in Sikkim. J Family Med Prim Care 8:988-994. https://doi.org/10.4103/jfmpc.jfmpc_32_19
220. Jamal SA, Arampatzis S, Harrison SL, Bucur RC, Ensrud K, Orwoll ES, Bauer DC (2015) Hyponatremia and Fractures: Findings From the MrOS Study. J Bone Miner Res 30:970-5. https://doi.org/10.1002/jbmr.2383
221. Jamil M, Ali U, Siraj J, Din IU, Ahmad T, Abbas M, Jan HU (2019) Frequency of hyponatremia and its short term clinical outcomes after acute ST elevation myocardial infarction. Pak. Heart J 52:307-312
222. Javed B, Fatima A, Ahmad, A (2018) Severity of Hyponatremia and its Influence on Various Complications Seen in Decompensated Chronic Liver Disease. Pak. J. Med. Health Sci 12:1221-1226
223. Jeon SB, Choi HA, Lesch C, Kim MC, Badjatia N, Claassen J, Mayer SA, Lee K (2013) Use of oral vasopressin V2 receptor antagonist for hyponatremia in acute brain injury. Eur Neurol 70:142-8. https://doi.org/10.1159/000350844
224. Jia JD, Xie W, Ding HG, Mao H, Guo H, Li Y, Wang X, Wang JF, Lu W, Li CZ, Mao Y, Wang GQ, Gao YQ, Wang B, Zhang Q, Ge Y, Wong VW (2017) Utility and safety of tolvaptan in cirrhotic patients with hyponatremia: A prospective cohort study. Ann Hepatol 16:123-132. https://doi.org/10.5604/16652681.1226823
225. Jonaidi Jafari N, Izadi M, Sarrafzadeh F, Heidari A, Ranjbar R, Saburi A (2013) Hyponatremia due to pulmonary tuberculosis: review of 200 cases. Nephrourol Mon 5:687-91. https://doi.org/10.5812/numonthly.7091
226. Jones BL, OʼHara JP, Till K, King RF (2015) Dehydration and hyponatremia in professional rugby union players: a cohort study observing english premiership rugby union players during match play, field, and gym training in cool environmental conditions. J Strength Cond Res 29:107-15. https://doi.org/10.1519/JSC.0000000000000620
227. Jong BH, Wei CC, Shyu KG (2016) Improved hyponatremia after pericardial drainage in patients suffering from cardiac tamponade. BMC Cardiovasc Disord 16:135. https://doi.org/10.1186/s12872-016-0316-1
228. Jönsson AK, Lövborg H, Lohr W, Ekman B, Rocklöv J (2017) Increased Risk of Drug- Induced Hyponatremia during High Temperatures. Int J Environ Res Public Health 14:827. https://doi.org/10.3390/ijerph14070827
229. Kagase A, Yamamoto M, Shimura T, Kodama A, Kano S, Koyama Y, Tada N, Naganuma T, Araki M, Yamanaka F, Shirai S, Watanabe Y, Hayashida (2018) Impact of pre-procedural hyponatremia on clinical outcomes after transcatheter aortic valve replacement: A propensity-matched analysis. Catheter Cardiovasc Interv 92:E125-E134. https://doi.org/10.1002/ccd.27483
230. Kajihara Y (2017) The prevalence and relevant factors of hyponatremia under long-term total enteral nutrition: A cross-sectional study. J Gen Fam Med 18:182-183. https://doi.org/10.1002/jgf2.44
231. Kala J, Mamlouk O, Jhaveri KD (2020) Selinexor-associated hyponatremia: single-center, real-world data. Kidney Int 98:789-791. https://doi.org/10.1016/j.kint.2020.06.007
232. Kalita J, Singh RK, Misra UK (2017) Cerebral Salt Wasting Is the Most Common Cause of Hyponatremia in Stroke. J Stroke Cerebrovasc Dis 26:1026-1032. https://doi.org/10.1016/j.jstrokecerebrovasdis.2016.12.011
233. Kanchanasurakit S, Saokaew S, Siriplabpla W, Arsu A, Boonmak W, Watcharasiriphong W (2020) Development of a hyponatremia screening tool (ABCDF-S score) for patients with hypertension using thiazide diuretic agents. J Clin Pharm Ther 45:997-1005. https://doi.org/10.1111/jcpt.13123
234. Karki L, Thapa B, Sah MK (2016) Hyponatremia in Patients with Community Acquired Pneumonia. JNMA J Nepal Med Assoc 54:67-71
235. Karunanandham S, Rajappa T, Selvaraju K (2018) Hyponatremia in Patients Admitted with Stroke. J. Clin. Diagn. Res 12:OC34-OC36
236. Käser SA, Furler R, Evequoz DC, Maurer CA (2013) Hyponatremia is a specific marker of perforation in sigmoid diverticulitis or appendicitis in patients older than 50 years. Gastroenterol Res Pract 2013:462891. https://doi.org/10.1155/2013/462891
237. Käser SA, Nitsche U, Maak M, Michalski CW, Späth C, Müller TC, Maurer CA, Janssen KP, Kleeff J, Friess H, Bader FG (2014) Could hyponatremia be a marker of anastomotic leakage after colorectal surgery? A single center analysis of 1,106 patients over 5 years. Langenbecks Arch Surg 399:783-8. https://doi.org/10.1007/s00423-014-1213-7
238. Kataoka Y, Nishida S, Hirakawa A, Oiso Y, Arima H (2015) Comparison of incidence of hyponatremia between intranasal and oral desmopressin in patients with central diabetes insipidus. Endocr J 62:195-200. https://doi.org/10.1507/endocrj.EJ14-0368
239. Katoch CD, Brar KS, Singh B (2013) Evaluation of thyroid and adrenal functions in patients with hyponatremia. Med J Armed Forces India 69:237-40. https://doi.org/10.1016/j.mjafi.2013.01.003
240. Kayar Y (2016) Evaluation of the frequency of hyponatremia and risk factors among hospitalized geriatric patients. Biomed. Res.-India 27:257-262
241. Khan FW, Fatima B, Lahr BD, Greason KL, Schaff HV, Dearani JA, Daly RC, Stulak JM, Crestanello JA (2021) Hyponatremia: An Overlooked Risk Factor Associated With Adverse Outcomes After Cardiac Surgery. Ann Thorac Surg 112:91-98. https://doi.org/10.1016/j.athoracsur.2020.08.030
242. Khow KS, Lau SY, Li JY, Yong TY (2014) Asymptomatic elevation of creatine kinase in patients with hyponatremia. Ren Fail 36:908-11. https://doi.org/10.3109/0886022X.2014.900600
243. Kim DY, Nassiri N, de Virgilio C, Ferebee MP, Kaji AH, Hamilton CE, Saltzman DJ (2015) Association Between Hyponatremia and Complicated Appendicitis. JAMA Surg 150:911-2. https://doi.org/10.1001/jamasurg.2015.1258
244. Kim HW, Ryu GW, Park CH, Kang EW, Park JT, Han SH, Yoo TH, Shin SK, Kang SW, Choi KH, Han DS, Chang TI (2015) Hyponatremia Predicts New-Onset Cardiovascular Events in Peritoneal Dialysis Patients. PLoS One 10:e0129480. https://doi.org/10.1371/journal.pone.0129480
245. Kim J, Cho SG, Kang SR, Kwon SY, Cho DH, Cho JS, Song HC (2017) Preparation for radioactive iodine therapy is not a risk factor for the development of hyponatremia in thyroid cancer patients. Medicine (Baltimore) 96:e6004. https://doi.org/10.1097/MD.0000000000006004
246. Kim JS, Lee JY, Park H, Han BG, Choi SO, Yang JW (2014) Estimation of body fluid volume by bioimpedance spectroscopy in patients with hyponatremia. Yonsei Med J 55:482-6. https://doi.org/10.3349/ymj.2014.55.2.482
247. Kim SE, Jung DM, Park JW, Ju Y, Lee B, Kim HS, Suk KT, Jang MK, Park SH, Kang JG, Soh JS, Lim H, Kang HS, Moon SH, Kim C, Lee S, Kim JH, Lee MS, Kim DJ, Ihm SH, Park C (2017) Baseline Renal Function Predicts Hyponatremia in Liver Cirrhosis Patients Treated with Terlipressin for Variceal Bleeding. Gastroenterol Res Pract 2017:7610374. https://doi.org/10.1155/2017/7610374
248. Kim Y, Lee N, Lee KE, Gwak HS (2020) Risk factors for sodium overcorrection in non-hypovolemic hyponatremia patients treated with tolvaptan. Eur J Clin Pharmacol 76:723-729. https://doi.org/10.1007/s00228-020-02848-6
249. Kim YS, Kim DW, Jung KH, Lee ST, Kang BS, Byun JI, Yeom JS, Chu K, Lee SK (2014) Frequency of and risk factors for oxcarbazepine-induced severe and symptomatic hyponatremia. Seizure 23:208-12. https://doi.org/10.1016/j.seizure.2013.11.015
250. Kleindienst A, Georgiev S, Schlaffer SM, Buchfelder M (2020) Tolvaptan Versus Fluid Restriction in the Treatment of Hyponatremia Resulting from SIADH Following Pituitary Surgery. J Endocr Soc 4:bvaa068. https://doi.org/10.1210/jendso/bvaa068
251. Kose SB, Hur E, Magden K, Yildiz G, Colak D, Kucuk E, Toka B, Kucuk H, Yildirim I, Kokturk F, Duman S (2015) Bioimpedance spectroscopy for the differential diagnosis of hyponatremia. Ren Fail 37:947-50. https://doi.org/10.3109/0886022X.2015.1040418
252. Krabak BJ, Lipman GS, Waite BL, Rundell SD (2017) Exercise-Associated Hyponatremia, Hypernatremia, and Hydration Status in Multistage Ultramarathons. Wilderness Environ Med 28:291-298. https://doi.org/10.1016/j.wem.2017.05.008
253. Kremeike K, Wetter RML, Burst V, Voltz R, Kuhr K, Simon ST (2018) Prevalence of hyponatremia in inpatients with incurable and life-limiting diseases and its association with physical symptoms-a retrospective descriptive study. Support Care Cancer 26:213-222. https://doi.org/10.1007/s00520-017-3837-y
254. Kriz J, Schuck O, Horackova M (2015) Hyponatremia in spinal cord injury patients: new insight into differentiating between the dilution and depletion forms. Spinal Cord 53:291-6. https://doi.org/10.1038/sc.2014.240
255. Kruse C, Eiken P, Verbalis J, Vestergaard P (2016) The effect of chronic mild hyponatremia on bone mineral loss evaluated by retrospective national Danish patient data. Bone 84:9-14. https://doi.org/10.1016/j.bone.2015.12.002
256. Kruse C, Eiken P, Vestergaard P (2015) Hyponatremia and osteoporosis: insights from the Danish National Patient Registry. Osteoporos Int 26:1005-16. https://doi.org/10.1007/s00198-014-2973-1
257. Kubota K, Sakaguchi Y, Hamano T, Oka T, Yamaguchi S, Shimada K, Matsumoto A, Hashimoto N, Mori D, Matsui I, Isaka Y (2020) Prognostic value of hypochloremia versus hyponatremia among patients with chronic kidney disease-a retrospective cohort study. Nephrol Dial Transplant 35:987-994. https://doi.org/10.1093/ndt/gfy299
258. Kuo SCH, Kuo PJ, Rau CS, Wu SC, Hsu SY, Hsieh CH (2017) Hyponatremia Is Associated with Worse Outcomes from Fall Injuries in the Elderly. Int J Environ Res Public Health 14:460. https://doi.org/10.3390/ijerph14050460
259. Kuramatsu JB, Bobinger T, Volbers B, Staykov D, Lücking H, Kloska SP, Köhrmann M, Huttner HB (2014) Hyponatremia is an independent predictor of in-hospital mortality in spontaneous intracerebral hemorrhage. Stroke 45:1285-91. https://doi.org/10.1161/STROKEAHA.113.004136
260. Kuryłowicz A, Wesołowska AS, Wolska A, Pachucki J, Bednarczuk T, Ambroziak U (2017) Safety and effectiveness of symptomatic hyponatremia treatment according to the European Society of Endocrinology guidelines: a retrospective study. Pol Arch Intern Med 127:205-208. https://doi.org/10.20452/pamw.3984
261. Kutz A, Ebrahimi F, Sailer CO, Wagner U, Schuetz P, Mueller B, Christ-Crain M (2020) Seasonality of Hypoosmolar Hyponatremia in Medical Inpatients - Data from a Nationwide Cohort Study. J Clin Endocrinol Metab 105:dgz320. https://doi.org/10.1210/clinem/dgz320
262. Lacquaniti A, Altavilla G, Picone A, Donato V, Chirico V, Mondello P, Aloisi C, Marabello G, Loddo S, Buemi A, Lorenzano G, Buemi M (2015) Apelin beyond kidney failure and hyponatremia: a useful biomarker for cancer disease progression evaluation. Clin Exp Med 15:97-105. https://doi.org/10.1007/s10238-014-0272-y
263. Lee A, Jo YH, Kim K, Ahn S, Oh YK, Lee H, Shin J, Chin HJ, Na KY, Lee JB, Baek SH, Kim S (2017) Efficacy and safety of rapid intermittent correction compared with slow continuous correction with hypertonic saline in patients with moderately severe or severe symptomatic hyponatremia: study protocol for a randomized controlled trial (SALSA trial). Trials 18:147. https://doi.org/10.1186/s13063-017-1865-z
264. Lee J, Kim DK, Lee JW, Oh KH, Oh YK, Na KY, Kim YS, Han JS, Suh KS, Joo KW (2013) Rapid correction rate of hyponatremia as an independent risk factor for neurological complication following liver transplantation. Tohoku J Exp Med 229:97-105. https://doi.org/10.1620/tjem.229.97
265. Lee JE, Kim SK, Han KH, Cho MO, Yun GY, Kim KH, Choi HY, Ryu YH, Ha SK, Park HC (2014) Risk factors for developing hyponatremia in thyroid cancer patients undergoing radioactive iodine therapy. PLoS One. 2014 9:e106840. https://doi.org/10.1371/journal.pone.0106840
266. Lee SW, Baek SH, Ahn SY, Na KY, Chae DW, Chin HJ, Kim S (2016) The Effects of Pre-Existing Hyponatremia and Subsequent-Developing Acute Kidney Injury on In-Hospital Mortality: A Retrospective Cohort Study. PLoS One 11:e0162990. https://doi.org/10.1371/journal.pone.0162990
267. Leth-Møller KB, Hansen AH, Torstensson M, Andersen SE, Ødum L, Gislasson G, Torp-Pedersen C, Holm EA (2016) Antidepressants and the risk of hyponatremia: a Danish register-based population study. BMJ Open 6:e011200. https://doi.org/10.1136/bmjopen-2016-011200
268. Levy-Shraga Y, David D, Vered I, Kochavi B, Stein D, Modan-Moses D (2016) Hyponatremia and decreased bone density in adolescent inpatients diagnosed with anorexia nervosa. Nutrition 32:1097-102. https://doi.org/10.1016/j.nut.2016.03.015
269. Lewis DP, Hoffman MD, Stuempfle KJ, Owen BE, Rogers IR, Verbalis JG, Hew-Butler TD (2014) The need for salt: does a relationship exist between cystic fibrosis and exercise-associated hyponatremia? J Strength Cond Res 28:807-13. https://doi.org/10.1519/JSC.0b013e3182a35dbd
270. Li F, Chen QX, Xiang SG, Yuan SZ, Xu XZ (2018) N-Terminal Pro-Brain Natriuretic Peptide Concentrations After Hypertensive Intracerebral Hemorrhage: Relationship With Hematoma Size, Hyponatremia, and Intracranial Pressure. J Intensive Care Med 33:663-670. https://doi.org/10.1177/0885066616683677
271. Li W, Chen X, Wang L, Wang Y, Huang C, Wang G, Du J (2019) The prognostic effects of hyponatremia and hyperchloremia on postoperative NSCLC patients. Curr Probl Cancer 43:402-410. https://doi.org/10.1016/j.currproblcancer.2018.12.006
272. Liao H, Zhu Z, Rong X, Wang H, Peng Y (2018) Hyponatremia is a potential predictor of progression in radiation-induced brain necrosis: a retrospective study. BMC Neurol 18:130. https://doi.org/10.1186/s12883-018-1135-z
273. Lim AKH, Paramaswaran S, Jellie LJ, Junckerstorff RK (2019) A Cross-Sectional Study of Hyponatremia Associated with Acute Central Nervous System Infections. J Clin Med 8:1801. https://doi.org/10.3390/jcm8111801
274. Lim CC, Siow B, Choo JCJ, Chawla M, Chin YM, Kee T, Lee PH, Foo M, Tan CS (2019) Desmopressin for the prevention of bleeding in percutaneous kidney biopsy: efficacy and hyponatremia. Int Urol Nephrol 51:995-1004. https://doi.org/10.1007/s11255-019-02155-9
275. Lim LM, Tsai NC, Lin MY, Hwang DY, Lin HY, Lee JJ, Hwang SJ, Hung CC, Chen HC (2016) Hyponatremia is Associated with Fluid Imbalance and Adverse Renal Outcome in Chronic Kidney Disease Patients Treated with Diuretics. Sci Rep 6:36817. https://doi.org/10.1038/srep36817
276. Lu DY, Cheng HM, Cheng YL, Hsu PF, Huang WM, Guo CY, Yu WC, Chen CH, Sung SH (2016) Hyponatremia and Worsening Sodium Levels Are Associated With Long-Term Outcome in Patients Hospitalized for Acute Heart Failure. J Am Heart Assoc 5:e002668. https://doi.org/10.1161/JAHA.115.002668
277. Lu H, Vollenweider P, Kissling S, Marques-Vidal P (2020) Prevalence and Description of Hyponatremia in a Swiss Tertiary Care Hospital: An Observational Retrospective Study. Front Med (Lausanne) 7:512. https://doi.org/10.3389/fmed.2020.00512
278. Mackanga JR, Dibambou N, Mouloungui EM, Iba-Ba J, Kombila UD, Bignoumba PI, Moussavou IM, Missounga L, Kombila JM, Boguikouma JB (2019) Intravenous infusion of glucose 5% despite sodium mixed, is main contributor of acquired hyponatremia in adult polyvalent medicine service: Case control study. Nephrol Ther 15:91-96. https://doi.org/10.1016/j.nephro.2018.10.004
279. MacMillan TE, Cavalcanti RB (2018) Outcomes in Severe Hyponatremia Treated With and Without Desmopressin. Am J Med 131:317.e1-317.e10. https://doi.org/10.1016/j.amjmed.2017.09.048
280. Madsen CM, Jantzen C, Lauritzen JB, Abrahamsen B, Jorgensen HL (2016) Hyponatremia and hypernatremia are associated with increased 30-day mortality in hip fracture patients. Osteoporos Int 27:397-404. https://doi.org/10.1007/s00198-015-3423-4
281. Maesaka JK, Imbriano LJ, Miyawaki N (2018) High Prevalence of Renal Salt Wasting Without Cerebral Disease as Cause of Hyponatremia in General Medical Wards. Am J Med Sci 356:15-22. https://doi.org/10.1016/j.amjms.2018.03.020
282. Mahesar SA, Memon SF, Mustafa S, Javed A, Butt SM (2019) Evaluation of Hyponatremia in Ischemic Stroke Patients in a Tertiary Care Hospital of Karachi, Pakistan. Cureus 11:e3926. https://doi.org/10.7759/cureus.3926
283. Maimaitili A, Maimaitili M, Rexidan A, Lu J, Ajimu K, Cheng X, Luo K, Sailike D, Liu Y, Kaheerman K, Tang C, Zhang T (2013) Pituitary hormone level changes and hypxonatremia in aneurysmal subarachnoid hemorrhage. Exp Ther Med 5:1657-1662. https://doi.org/10.3892/etm.2013.1068
284. Malabu UH, Porter D, Vangaveti VN, Kazi M, Kennedy RL (2014) Prevalence of hyponatremia in acute medical admissions in tropical Asia Pacific Australia. Asian Pac J Trop Med 7:40-3. https://doi.org/10.1016/S1995-7645(13)60189-3
285. Mannesse CK, Jansen PA, Van Marum RJ, Sival RC, Kok RM, Haffmans PM, Egberts TC (2013) Characteristics, prevalence, risk factors, and underlying mechanism of hyponatremia in elderly patients treated with antidepressants: a cross-sectional study. Maturitas 76:357-63. https://doi.org/10.1016/j.maturitas.2013.08.010
286. Mannheimer B, Skov J, Falhammar H, Calissendorff J, Lindh JD, Nathanson D (2019) Sex-specific risks of death in patients hospitalized for hyponatremia: a population-based study. Endocrine 66:660-665. https://doi.org/10.1007/s12020-019-02073-x
287. Mansoor A, Ather CAA, Hameed B, Khosa M, Kahn IM, Khalid R (2017) Association of Mortality with Hyponatremia in patients of Heart Failure. Pak. J. Med. Health Sci 11:988-991
288. Marco J, Barba R, Matía P, Plaza S, Méndez M, Canora J, Zapatero A (2013) Low prevalence of hyponatremia codification in departments of internal medicine and its prognostic implications. Curr Med Res Opin 29:1757-62. https://doi.org/10.1185/03007995.2013.836079
289. Marr N, Yu J, Kutsogiannis DJ, Mahmoud SH (2017) Risk of Hyponatremia in Patients with Aneurysmal Subarachnoid Hemorrhage Treated with Exogenous Vasopressin Infusion. Neurocrit Care 26:182-190. https://doi.org/10.1007/s12028-016-0300-8
290. Martin JY, Goff BA, Urban RR (2016) Preoperative hyponatremia in women with ovarian cancer: An additional cause for concern? Gynecol Oncol 142:471-6. https://doi.org/10.1016/j.ygyno.2016.06.018
291. Maruyama H, Kondo T, Sekimoto T, Kiyono S, Shimada T, Takahashi M, Okugawa H, Yokosuka O (2015) Hyponatremia: a significant factor in a poor prognosis for cirrhosis with Child A/B after variceal eradication. J Hepatobiliary Pancreat Sci 22:771-8. https://doi.org/10.1002/jhbp.282
292. Matro R, Daskalakis C, Negoianu D, et al (2014) Randomised clinical trial: Polyethylene glycol 3350 with sports drink vs. polyethylene glycol with electrolyte solution as purgatives for colonoscopy - The incidence of hyponatraemia. Aliment Pharmacol Ther 40:610–619. https://doi.org/10.1111/apt.12884
293. Mayer CU, Treff G, Fenske WK, Blouin K, Steinacker JM, Allolio B (2015) High incidence of hyponatremia in rowers during a four-week training camp. Am J Med 128:1144-51. https://doi.org/10.1016/j.amjmed.2015.04.014
294. Méndez-Bailón M, Barba-Martín R, de Miguel-Yanes JM, Zapatero-Gaviria A, Calvo-Porqueras B, Osuna MF, Nuñez-Fernández C, Muñoz-Rivas N, Plaza Canteli S, Marco-Martínez J (2015) Hyponatremia in hospitalised patients with heart failure in internal medicine: Analysis of the Spanish national minimum basic data set (MBDS) (2005-2011). Eur J Intern Med 26:603-6. https://doi.org/10.1016/j.ejim.2015.06.009
295. Meng X, Shi B (2016) Traumatic Brain Injury Patients With a Glasgow Coma Scale Score of ≤8, Cerebral Edema, and/or a Basal Skull Fracture are More Susceptible to Developing Hyponatremia. J Neurosurg Anesthesiol 28:21-6. https://doi.org/10.1097/ANA.0000000000000192
296. Merola J, Chaudhary N, Qian M, Jow A, Barboza K, Charles H, Teperman L, Sigal S (2014) Hyponatremia: A Risk Factor for Early Overt Encephalopathy after Transjugular Intrahepatic Portosystemic Shunt Creation. J Clin Med 3:359-72. https://doi.org/10.3390/jcm3020359
297. Michal O, Magdalena MZ, Halina M, Magdalena BZ, Tadeusz N, Elzbieta M, Marcin W (2016) Hyponatremia effect in patients with alcohol dependence on their physical and mental health status. Alcohol 57:49-53. https://doi.org/10.1016/j.alcohol.2016.10.002
298. Miles JA, Quispe R, Mehlman Y, Patel K, Lama Von Buchwald C, You JY, Sokol S, Faillace RT (2019) Racial differences and mortality risk in patients with heart failure and hyponatremia. PLoS One 14:e0218504. https://doi.org/10.1371/journal.pone.0218504
299. Miljic D, Doknic M, Stojanovic M, Nikolic-Djurovic M, Petakov M, Popovic V, Pekic S (2017) Impact of etiology, age and gender on onset and severity of hyponatremia in patients with hypopituitarism: retrospective analysis in a specialised endocrine unit. Endocrine 58:312-319. https://doi.org/10.1007/s12020-017-1415-1
300. Miller WL, Grill DE, Struck J, Jaffe AS (2013) Association of hyponatremia and elevated copeptin with death and need for transplantation in ambulatory patients with chronic heart failure. Am J Cardiol 111:880-5. https://doi.org/10.1016/j.amjcard.2012.11.053
301. Misra UK, Kalita J, Bhoi SK, Singh RK (2016) A study of hyponatremia in tuberculous meningitis. J Neurol Sci 367:152-7. https://doi.org/10.1016/j.jns.2016.06.004
302. Misra UK, Kalita J, Singh RK, Bhoi SK (2019) A Study of Hyponatremia in Acute Encephalitis Syndrome: A Prospective Study From a Tertiary Care Center in India. J Intensive Care Med 34:411-417. https://doi.org/10.1177/0885066617701422
303. Mohan S, Gu S, Parikh A, Radhakrishnan J (2013) Prevalence of hyponatremia and association with mortality: results from NHANES. Am J Med 126:1127-37.e1. https://doi.org/10.1016/j.amjmed.2013.07.021
304. Morris JH, Bohm NM, Nemecek BD, Crawford R, Kelley D, Bhasin B, Nietert PJ, Velez JCQ (2018) Rapidity of Correction of Hyponatremia Due to Syndrome of Inappropriate Secretion of Antidiuretic Hormone Following Tolvaptan. Am J Kidney Dis 71:772-782. https://doi.org/10.1053/j.ajkd.2017.12.002
305. Mrowczynski OD, Bourcier AJ, Liao J, Langan ST, Specht CS, Rizk EB (2018) The predictive potential of hyponatremia for glioblastoma patient survival. J Neurooncol 138:99-104. https://doi.org/10.1007/s11060-018-2774-z
306. Mumtaz M, Ahmad W, Khan AH (2017) Frequency of Hyponatremia in patients of Chronic Liver Disease. Pak. J. Med. Health Sci 11:1214-1216
307. Murakami K, Kohashi S, Sakurai M, Kato J, Toyama T, Koda Y, Yamane Y, Hashida R, Abe R, Yamazaki R, Kikuchi T, Shimizu T, Suzuki S, Hasegawa N, Okamoto S, Mori T (2017) Hyponatremia associated with human herpesvirus-6 (HHV-6) encephalitis after allogeneic hematopoietic stem cell transplantation: A presentation different from HHV-6 myelitis. Int J Hematol 106:436-440. https://doi.org/10.1007/s12185-017-2254-9
308. Murthy K, Koshkina O, Marcantonio AJ, Pala N, Breeze JL, Paulus J, Hodge MB (2015) Hyponatremia and Fracture Risk: A Hospital-Based Case--Control Study. J Am Geriatr Soc 63:1699-701. https://doi.org/10.1111/jgs.13573
309. Musch W, Decaux G (2018) Severe Solute Depletion in Patients with Hyponatremia Due to Diuretics Despite Biochemical Pictures Similar Than Those Observed in the Syndrome of Inappropriate Secretion of Antidiuretic Hormone. Nephron 140:31-38. https://doi.org/10.1159/000490203
310. Nagata T, Nakajima S, Fujiya A, Sobajima H, Yamaguchi M (2018) Prevalence of hypothyroidism in patients with hyponatremia: A retrospective cross-sectional study. PLoS One 13:e0205687. https://doi.org/10.1371/journal.pone.0205687
311. Nair S, Mary TR, Tarey SD, Daniel SP, Austine J (2016) Prevalence of Hyponatremia in Palliative Care Patients. Indian J Palliat Care 22:33-7. https://doi.org/10.4103/0973-1075.173954
312. Naka T, Kohagura K, Kochi M, Ohya Y (2018) Hyponatremia and mortality among very elderly residents in a geriatric health service facility. Clin Exp Nephrol 22:1404-1410. https://doi.org/10.1007/s10157-018-1607-x
313. Nervo A, D'Angelo V, Rosso D, Castellana E, Cattel F, Arvat E, Grossi E (2019) Urea in cancer patients with chronic SIAD-induced hyponatremia: Old drug, new evidence. Clin Endocrinol (Oxf) 90:842-848. https://doi.org/10.1111/cen.13966
314. Ng TM, Cao DX, Patel KA, Wong YM, Prasad M, Lou M, Elkayam U (2014) Association of hyponatremia to diuretic response and incidence of increased serum creatinine levels in hospitalized patients with acute decompensated heart failure. Cardiology 128:333-42. https://doi.org/10.1159/000360604
315. Ng TMH, Grazette LP, Fong MW, Yoon AJ, Lou M, Kuo A, Upadhyay RY, Han EE, Mehra A, Elkayam U (2020) Tolvaptan vs. furosemide-based diuretic regimens in patients hospitalized for heart failure with hyponatremia (AQUA-AHF). ESC Heart Fail 7:1927-1934. https://doi.org/10.1002/ehf2.12783
316. Nigro N, Winzeler B, Suter-Widmer I, et al (2015) Mid-regional pro-atrial natriuretic peptide and the assessment of volaemic status and differential diagnosis of profound hyponatraemia. J Intern Med (GBR) 278:29–37. https://doi.org/10.1111/joim.12332
317. Nigro N, Winzeler B, Suter-Widmer I, et al (2017) Evaluation of copeptin and commonly used laboratory parameters for the differential diagnosis of profound hyponatraemia in hospitalized patients: ‘The Co-MED Study’. Clin Endocrinol 86:456–462. https://doi.org/10.1111/cen.13243
318. Nigro N, Winzeler B, Suter-Widmer I, Schuetz P, Arici B, Bally M, Blum C, Bingisser R, Bock A, Huber A, Müller B, Nickel CH, Christ-Crain M (2015) Symptoms and characteristics of individuals with profound hyponatremia: a prospective multicenter observational study. J Am Geriatr Soc 63:470-5. https://doi.org/10.1111/jgs.13325
319. Nigwekar SU, Negri AL, Bajpai D, Allegretti A, Kalim S, Seethapathy H, Bhan I, Murthy K, Ayus JC (2019) Chronic prolonged hyponatremia and risk of hip fracture in elderly patients with chronic kidney disease. Bone 127:556-562. https://doi.org/10.1016/j.bone.2019.07.029
320. Nigwekar SU, Wenger J, Thadhani R, Bhan I (2013) Hyponatremia, mineral metabolism, and mortality in incident maintenance hemodialysis patients: a cohort study. Am J Kidney Dis 62:755-62. https://doi.org/10.1053/j.ajkd.2013.02.367
321. Nishi Y, Ogami C, Tsuji Y, Kawasuji H, Yamada H, Kawai S, Sakamaki I, To H, Yamamoto Y (2021) Evaluation of the relationship between linezolid exposure and hyponatremia. J Infect Chemother 27:165-171. https://doi.org/10.1016/j.jiac.2020.08.017
322. Nishikawa H, Kita R, Kimura T, Ohara Y, Sakamoto A, Saito S, Nishijima N, Nasu A, Komekado H, Osaki Y (2015) Hyponatremia in hepatocellular carcinoma complicating with cirrhosis. J Cancer 6:482-9. https://doi.org/10.7150/jca.11665
323. Nolte HW, Nolte K, Hew-Butler T (2019) Ad libitum water consumption prevents exercise-associated hyponatremia and protects against dehydration in soldiers performing a 40-km route-march. Mil Med Res 6:1. https://doi.org/10.1186/s40779-019-0192-y
324. Ochi N, Yamane H, Hotta K, Fujii H, Isozaki H, Honda Y, Yamagishi T, Kubo T, Tanimoto M, Kiura K, Takigawa N (2014) Cisplatin-induced hyponatremia in malignancy: comparison between brand-name and generic formulation. Drug Des Devel Ther 8:2401-8. https://doi.org/10.2147/DDDT.S71419
325. Oh J, Kang SM, Kim IC, Han S, Yoo BS, Choi DJ, Kim JJ, Jeon ES, Cho MC, Oh BH, Chae SC, Lee MM, Ryu KH (2017) The beneficial prognostic value of hemoconcentration is negatively affected by hyponatremia in acute decompensated heart failure: Data from the Korean Heart Failure (KorHF) Registry. J Cardiol 69:790-796. https://doi.org/10.1016/j.jjcc.2016.08.003
326. Ohbe H, Koakutsu T, Kushimoto S (2019) Analysis of risk factors for hyponatremia in patients with acute spinal cord injury: a retrospective single-institution study in Japan. Spinal Cord 57:240-246. https://doi.org/10.1038/s41393-018-0208-6
327. Okada M, Egi M, Yokota Y, Shirakawa N, Fujimoto D, Taguchi S, Furushima N, Mizobuchi S (2017) Comparison of the incidences of hyponatremia in adult postoperative critically ill patients receiving intravenous maintenance fluids with 140 mmol/L or 35 mmol/L of sodium: retrospective before/after observational study. J Anesth 31:657-663. https://doi.org/10.1007/s00540-017-2370-y
328. Olin JL, Mitchell G, Cremisi H (2015) Experience with Tolvaptan for Euvolemic and Hypervolemic Hyponatremia in the Acute Care Setting. Hosp Pharm 50:380-5. https://doi.org/10.1310/hpj5005-380
329. Omar HR, Charnigo R, Guglin M (2017) Prognostic Significance of Discharge Hyponatremia in Heart Failure Patients With Normal Admission Sodium (from the ESCAPE Trial). Am J Cardiol 120:607-615. https://doi.org/10.1016/j.amjcard.2017.05.030
330. Omar HR, Guglin M (2016) Community acquired versus hospital acquired hyponatremia in acute heart failure: Association with clinical characteristics and outcomes. Int J Cardiol 225:247-249. https://doi.org/10.1016/j.ijcard.2016.09.135
331. Omar HR, Guglin M (2018) Etiology of discharge hyponatremia in decompensated heart failure and normal admission Na<sup>+</sup>: Effect of diuretics. Eur J Intern Med 48:e15-e17. https://doi.org/10.1016/j.ejim.2017.12.007
332. Oude Lansink-Hartgring A, Hessels L, Weigel J, de Smet AMGA, Gommers D, Panday PVN, Hoorn EJ, Nijsten MW (2016) Long-term changes in dysnatremia incidence in the ICU: a shift from hyponatremia to hypernatremia. Ann Intensive Care 6:22. https://doi.org/10.1186/s13613-016-0124-x
333. Ouellette L, Michel K, Riley B, Jones J (2018) Beer potomania: Atypical cause of severe hyponatremia in older alcoholics. Am J Emerg Med 36:1303. https://doi.org/10.1016/j.ajem.2017.10.065
334. Owen BE, Rogers IR, Hoffman MD, Stuempfle KJ, Lewis D, Fogard K, Verbalis JG, Hew-Butler T (2014) Efficacy of oral versus intravenous hypertonic saline in runners with hyponatremia. J Sci Med Sport 17:457-62. https://doi.org/10.1016/j.jsams.2013.09.001
335. Ozdemir E, Polat SB, Talay NB (2020) Do Patients with Differentiated Thyroid Cancer Face the Risk of Hyponatremia at the Expense of Preparation for Radioactive Iodine Treatment?. Eur. J. Ther 26:233-237
336. Padhi R, Panda BN, Jagati S, Patra SC (2014) Hyponatremia in critically ill patients. Indian J Crit Care Med 18:83-7. https://doi.org/10.4103/0972-5229.126077
337. Palmer BF, Rock AD, Woodward EJ (2016) Dose comparison of conivaptan (Vaprisol®) in patients with euvolemic or hypervolemic hyponatremia--efficacy, safety, and pharmacokinetics. Drug Des Devel Ther 10:339-51. https://doi.org/10.2147/DDDT.S95326
338. Pan X, Zhou Z, Jin X, Shi D (2020) Clinical characteristics and risk factors of severe hyponatremia in cirrhotic patients treated with terlipressin. J Clin Pharm Ther 45:191-198. https://doi.org/10.1111/jcpt.13057
339. Park JJ, Cho YJ, Oh IY, Park HA, Lee HY, Kim KH, Yoo BS, Kang SM, Baek SH, Jeon ES, Kim JJ, Cho MC, Chae SC, Oh BH, Choi DJ (2017) Short and long-term prognostic value of hyponatremia in heart failure with preserved ejection fraction versus reduced ejection fraction: An analysis of the Korean Acute Heart Failure registry. Int J Cardiol 248:239-245. https://doi.org/10.1016/j.ijcard.2017.08.004
340. Park M, Son HJ, Kim GS (2019) Osmotic Demyelination Syndrome Following Hyponatremia-Oriented Management in Liver Transplant: A Single Center 20-Year Experience. Exp Clin Transplant 17:540-545. https://doi.org/10.6002/ect.2018.0216
341. Park S, An JN, Lee JP, Oh YK, Kim DK, Joo KW, Kim YS, Lim CS (2017) Association between postoperative hyponatremia and renal prognosis in major urologic surgery. Oncotarget 8:79935-79947. https://doi.org/10.18632/oncotarget.20326
342. Patel KS, Shu Chen J, Yuan F, Attiah M, Wilson B, Wang MB, Bergsneider M, Kim W (2019) Prediction of post-operative delayed hyponatremia after endoscopic transsphenoidal surgery. Clin Neurol Neurosurg 182:87-91. https://doi.org/10.1016/j.clineuro.2019.05.007
343. Patel S, Chiu RG, Rosinski CL, Chaker AN, Burch TG, Behbahani M, Sadeh M, Mehta AI (2020) Risk Factors for Hyponatremia and Perioperative Complications With Malignant Intracranial Tumor Resection in Adults: An Analysis of the Nationwide Inpatient Sample from 2012 to 2015. World Neurosurg 144:e876-e882. https://doi.org/10.1016/j.wneu.2020.09.097
344. Patel S, Nguyen DS, Rastogi A, Nguyen MK, Nguyen MK (2017) Treatment of Cirrhosis- Associated Hyponatremia with Midodrine and Octreotide. Front Med (Lausanne) 4:17. https://doi.org/10.3389/fmed.2017.00017
345. Patil VC, Patil HV, Malakar IB (2019) Clinical profile and outcome of patients with hyponatremia at tertiary care teaching hospital. J. Evol. Med. Dent. Sci 7:5591-5596
346. Patra S, Kumar B, Harlalka KK, Jain A, Bhanuprakash HM, Sadananda KS, Basappa H, Santhosh K, Rajith KS, Bharathi KS, Manjunath CN (2014) Short term efficacy and safety of low dose tolvaptan in patients with acute decompensated heart failure with hyponatremia: a prospective observational pilot study from a single center in South India. Heart Views 15:1-5. https://doi.org/10.4103/1995-705X.132136
347. Pedersen MM, Donskov F, Pedersen L, Zhang ZF, Nørgaard M (2020) Elevated neutrophil-lymphocyte ratio combined with hyponatremia indicate poor prognosis in renal cell carcinoma. Acta Oncol 59:13-19. https://doi.org/10.1080/0284186X.2019.1654128
348. Pennington Z, Bomberger TT, Lubelski D, Benzel EC, Steinmetz MP, Mroz TE (2020) Preoperative Hyponatremia and Perioperative Complications in Cervical Spinal Fusion. World Neurosurg 141:e864-e872. https://doi.org/10.1016/j.wneu.2020.06.068
349. Penttilä P, Bono P, Peltola K, Donskov F (2018) Hyponatremia associates with poor outcome in metastatic renal cell carcinoma patients treated with everolimus: prognostic impact. Acta Oncol 57:1580-1585. https://doi.org/10.1080/0284186X.2018.1477256
350. Pereira G, Baldin C, Piedade J, Reis V, Valdeolivas T, Victor L, Guimarães L, Duarte J, Veiga Z, Alcântara C, Fernandes F, Pereira JL (2020) Combination and sequential evaluation of acute-on-chronic liver failure (ACLF) and hyponatremia and prognosis in cirrhotic patients. Dig Liver Dis 52:91-97. https://doi.org/10.1016/j.dld.2019.08.013
351. Petereit C, Zaba O, Teber I, Lüders H, Grohé C (2013) A rapid and efficient way to manage hyponatremia in patients with SIADH and small cell lung cancer: treatment with tolvaptan. BMC Pulm Med 13:55. https://doi.org/10.1186/1471-2466-13-55
352. Peyro Saint Paul L, Martin J, Buon M, Gaillard C, Fedrizzi S, Mosquet B, Coquerel A (2014) Nouvel effet indésirable fréquent des inhibiteurs de la pompe à protons chez le sujet âgé : l'hyponatrémie modérée [New frequent adverse reaction of PPI in older adults: mild hyponatremia]. Therapie 69:157-62. https://doi.org/10.2515/therapie/2014019
353. Peyro Saint Paul L, Martin J, Gaillard C, Mosquet B, Coquerel A, de la Gastine B (2013) L'hyponatrémie modérée potentiellement médicamenteuse du sujet âgé: bénéfice de la réduction des médicaments [Moderate potentially drug-induced hyponatremia in older adults: benefit in drug reduction]. Therapie 68:341-6. French. https://doi.org/10.2515/therapie/2013058
354. Pliquett RU, Schlump K, Wienke A, Bartling B, Noutsias M, Tamm A, Girndt M (2020) Diabetes prevalence and outcomes in hospitalized cardiorenal-syndrome patients with and without hyponatremia. BMC Nephrol 21:393. https://doi.org/10.1186/s12882-020-02032-z
355. Potasso L, Sailer CO, Blum CA, Cesana-Nigro N, Schuetz P, Mueller B, Christ-Crain M (2020) Mild to moderate hyponatremia at discharge is associated with increased risk of recurrence in patients with community-acquired pneumonia. Eur J Intern Med 75:44-49. https://doi.org/10.1016/j.ejim.2019.12.009
356. Prempunpong C, Efanov I, Sant'anna G (2013) The effect of the implementation of therapeutic hypothermia on fluid balance and incidence of hyponatremia in neonates with moderate or severe hypoxic-ischaemic encephalopathy. Acta Paediatr 102:e507-13. https://doi.org/10.1111/apa.12362
357. Qiu Y, Ye H, Wang Y, Zhong Z, Li H, Huang F, Yang X, Yu X, Mao H (2020) Age Difference in the Association between Hyponatremia and Infection-Related Mortality in Peritoneal Dialysis Patients. Blood Purif 49:631-640. https://doi.org/10.1159/000505614
358. Quinn L, Tian DH, Fitzgerald E, Flower O, Andersen C, Hammond N, Davidson K, Delaney A (2020) The association between hyponatraemia and long-term functional outcome in patients with aneurysmal subarachnoid haemorrhage: A single centre prospective cohort study. J Clin Neurosci 78:353-359. https://doi.org/10.1016/j.jocn.2020.06.003
359. Qureshi MAM, Zubair N, Rafiq M (2017) Frequency of Hyponatremia in Patients with Liver Cirrhosis. Pak. J. Med. Health Sci 11:562-565
360. Qureshi MO, Khokhar N, Saleem A, Niazi TK (2014) Correlation of hyponatremia with hepatic encephalopathy and severity of liver disease. J Coll Physicians Surg Pak 24:135-7
361. Qureshi SS, Amer W, Javaid AM, Awais M, Kazmi K (2020) Hyponatremia - A Risk Factor for Mortality in Cirrhotics. Pak. J. Med. Health Sci 14:679-682
362. Qureshi W, Hassan S, Khalid F, Almahmoud MF, Shah B, Tashman R, Ambulgekar N, El-Refai M, Mittal C, Alirhayim Z (2013) Outcomes of correcting hyponatremia in patients with myocardial infarction. Clin Res Cardiol 102:637-44. https://doi.org/10.1007/s00392-013-0576-z
363. Rabinovitz A, Raiszadeh F, Zolty R (2013) Association of hyponatremia and outcomes in pulmonary hypertension. J Card Fail 19:550-6. https://doi.org/10.1016/j.cardfail.2013.05.014
364. Rafat C, Schortgen F, Gaudry S, Bertrand F, Miguel-Montanes R, Labbé V, Ricard JD, Hajage D, Dreyfuss D (2014) Use of desmopressin acetate in severe hyponatremia in the intensive care unit. Clin J Am Soc Nephrol 9:229-37. https://doi.org/10.2215/CJN.00950113
365. Rajagopal R, Swaminathan G, Nair S, Joseph M (2017) Hyponatremia in Traumatic Brain Injury: A Practical Management Protocol. World Neurosurg 108:529-533. https://doi.org/10.1016/j.wneu.2017.09.013
366. Rajan S, Srikumar S, Paul J, Kumar L (2015) Effectiveness of single dose conivaptan for correction of hyponatraemia in post-operative patients following major head and neck surgeries. Indian J Anaesth 59:416–420. https://doi.org/10.4103/0019-5049.160943
367. Rajan S, Tosh P, Kadapamannil D, Srikumar S, Paul J, Kumar L (2018) Efficacy of vaptans for correction of postoperative hyponatremia: A comparison between single intravenous bolus conivaptan vs oral tolvaptan. J Anaesthesiol Clin Pharmacol 34:193-197. https://doi.org/10.4103/joacp.JOACP_263_17
368. Ramberg E, Greve AM, Berg RMG, Sajadieh A, Haugaard SB, Willenheimer R, Olsen MH, Wachtell K, Nielsen OW (2021) Frequency and Impact of Hyponatremia on All-Cause Mortality in Patients With Aortic Stenosis. Am J Cardiol 141:93-97. https://doi.org/10.1016/j.amjcard.2020.11.015
369. Ramírez E, Rodríguez A, Queiruga J, García I, Díaz L, Martínez L, Muñoz R, Muñoz M, Tong HY, Martínez JC, Borobia AM, Carcas AJ, Frías J (2019) Severe Hyponatremia Is Often Drug Induced: 10-Year Results of a Prospective Pharmacovigilance Program. Clin Pharmacol Ther 106:1362-1379. https://doi.org/10.1002/cpt.1562
370. Rani RU, Sameera B(2020) A Clinical and Aetiological Study of Hyponatremia in Patients Admitted to Medical Intensive Care Unit in a Rural Tertiary Care Hospital. J. Evol. Med. Dent. Sci 9:1341-1344
371. Rasheeq T, Ahmed M, Ather MM, Furqan A (2019) Hyponatremia as a predicting factor of mortality in chronic liver disease. Rawal Med. J 44:440-444
372. Razavi AS, Chasen ST, Gyawali R, Kalish RB (2017) Hyponatremia associated with preeclampsia. J Perinat Med 45:467-470. https://doi.org/10.1515/jpm-2016-0062
373. Rech JS, Yao K, Bachmeyer C, Bailleul S, Javier O, Grateau G, Lionnet F, Steichen O (2020) Prognostic Value of Hyponatremia During Acute Painful Episodes in Sickle Cell Disease. Am J Med 133:e465-e482. https://doi.org/10.1016/j.amjmed.2020.02.017
374. Reddy SN, Rangappa P, Jacob I, Janakiraman R, Rao K (2016) Efficacy of conivaptan and hypertonic (3%) saline in treating hyponatremia due to syndrome of inappropriate antidiuretic hormone in a tertiary Intensive Care Unit. Indian J Crit Care Med 20:714-718. https://doi.org/10.4103/0972-5229.195708
375. Refardt J, Kling B, Krausert K, Fassnacht M, von Felten S, Christ-Crain M, Fenske W (2018) Impact of chronic hyponatremia on neurocognitive and neuromuscular function. Eur J Clin Invest 48:e13022. https://doi.org/10.1111/eci.13022
376. Renneboog B, Sattar L, Decaux G (2017) Attention and postural balance are much more affected in older than in younger adults with mild or moderate chronic hyponatremia. Eur J Intern Med 41:e25-e26. https://doi.org/10.1016/j.ejim.2017.02.008
377. Ridwan S, Zur B, Kurscheid J, Esche J, Kristof R, Klingmüller D, Boström A (2019) Hyponatremia After Spontaneous Aneurysmal Subarachnoid Hemorrhage-A Prospective Observational Study. World Neurosurg 129:e538-e544. https://doi.org/10.1016/j.wneu.2019.05.210
378. Rinaldi S, Santoni M, Leoni G, Fiordoliva I, Marcantognini G, Meletani T, Armento G, Santini D, Newsom-Davis T, Tiberi M, Morgese F, Torniai M, Bower M, Berardi R (2019) The prognostic and predictive role of hyponatremia in patients with advanced non-small cell lung cancer (NSCLC) with bone metastases. Support Care Cancer 27:1255-1261. https://doi.org/10.1007/s00520-018-4489-2
379. Rittenhouse KJ, To T, Rogers A, Wu D, Horst M, Edavettal M, Miller JA, Rogers FB (2015) Hyponatremia as a fall predictor in a geriatric trauma population. Injury 46:119-23. https://doi.org/10.1016/j.injury.2014.06.013
380. Rocha AFB, Sá MVBO, Elihimas Junior UF (2019) Hyponatremia in elderly patients with fragility fractures of the proximal femur: a cross-sectional study. J Bras Nefrol 41:518-525. https://doi.org/10.1590/2175-8239-JBN-2019-0019
381. Rodenburg EM, Hoorn EJ, Ruiter R, Lous JJ, Hofman A, Uitterlinden AG, Stricker BH, Visser LE (2013) Thiazide-associated hyponatremia: a population-based study. Am J Kidney Dis 62:67-72. https://doi.org/10.1053/j.ajkd.2013.02.365
382. Rodrigues B, Staff I, Fortunato G, McCullough LD (2014) Hyponatremia in the prognosis of acute ischemic stroke. J Stroke Cerebrovasc Dis 23:850-4. https://doi.org/10.1016/j.jstrokecerebrovasdis.2013.07.011
383. Rodríguez-de Muñoz YM, Sánchez-Lázaro IJ, Almenar-Bonet L, Martínez-Dolz L, Rodríguez-Serrano M, Salvador-Sanz A (2013) Use of tolvaptan in patients with hyponatremia due to heart failure: initial experience. Rev Esp Cardiol (Engl Ed) 66:319-21. https://doi.org/10.1016/j.rec.2012.09.010
384. Rondon-Berrios H, Tandukar S, Mor MK, Ray EC, Bender FH, Kleyman TR, Weisbord SD (2018) Urea for the Treatment of Hyponatremia. Clin J Am Soc Nephrol 13:1627-1632. https://doi.org/10.2215/CJN.04020318
385. Rudkovskaia AA, Tonelli AR, Rao Y, Hammel JP, Buller GK, Dweik RA, Fares WH (2018) Is hyponatremia associated with mortality in pulmonary arterial hypertension? Pulm Circ 8:2045894018776888. https://doi.org/10.1177/2045894018776888
386. Ruiz-Sánchez JG, Meneses D, Álvarez-Escolá C, Cuesta M, Calle-Pascual AL, Runkle I (2020) The Effect of the Dose of Isotonic Saline on the Correction of Serum Sodium in the Treatment of Hypovolemic Hyponatremia. J Clin Med 9:3567. https://doi.org/10.3390/jcm9113567
387. Ruiz-Sánchez JG, Núñez-Gil IJ, Cuesta M, Rubio MA, Maroun-Eid C, Arroyo-Espliguero R, Romero R, Becerra-Muñoz VM, Uribarri A, Feltes G, Trabattoni D, Molina M, García Aguado M, Pepe M, Cerrato E, Alfonso E, Castro Mejía AF, Roubin SR, Buzón L, Bondia E, Marin F, López Pais J, Abumayyaleh M, D'Ascenzo F, Rondano E, Huang J, Fernandez-Perez C, Macaya C, de Miguel Novoa P, Calle-Pascual AL, Estrada Perez V, Runkle I; HOPE COVID-19 investigators (2020) Prognostic Impact of Hyponatremia and Hypernatremia in COVID-19 Pneumonia. A HOPE-COVID-19 (Health Outcome Predictive Evaluation for COVID-19) Registry Analysis. Front Endocrinol (Lausanne) 11:599255. https://doi.org/10.3389/fendo.2020.599255
388. Rumalla K, Reddy AY, Letchuman V, Mittal MK (2017) Hyponatremia in Guillain-Barré Syndrome. J Clin Neuromuscul Dis 18:207-217. https://doi.org/10.1097/CND.0000000000000157
389. Ruocco G, Verbrugge FH, Nuti R, Palazzuoli A (2018) Hyponatremia in Acute Heart Failure in Relation to Hematocrit Levels: Clinical Relevance and Prognostic Implication. Cardiorenal Med 8:259-270. https://doi.org/10.1159/000490767
390. Ruzicka M, McCormick B, Magner P, Ramsay T, Edwards C, Bugeja A, Hiremath S (2018) Thiazide diuretic-caused hyponatremia in the elderly hypertensive: will a bottle of Nepro a day keep hyponatremia and the doctor away? Study protocol for a proof-of-concept feasibility trial. Pilot Feasibility Stud 4:71. https://doi.org/10.1186/s40814-018-0263-y
391. Saepudin S, Ball PA, Morrissey H (2015) Hyponatremia during hospitalization and in-hospital mortality in patients hospitalized from heart failure. BMC Cardiovasc Disord 15:88. https://doi.org/10.1186/s12872-015-0082-5
392. Saepudin S, Ball PA, Morrissey H (2016) Patient and medication-related factors associated with hospital-acquired hyponatremia in patients hospitalized from heart failure. Int J Clin Pharm 38:848-54. https://doi.org/10.1007/s11096-016-0296-3
393. Sağ S, Aydın Kaderli A, Yıldız A, Gül BC, Özdemir B, Baran İ, Güllülü S, Aydınlar A, Çavuşoğlu Y (2017) Use of tolvaptan in patients hospitalized for worsening chronic heart failure with severe hyponatremia: The initial experience at a single-center in Turkey. Turk Kardiyol Dern Ars 45:415-425. https://doi.org/10.5543/tkda.2017.80026
394. Sailer CO, Winzeler B, Nigro N, Bernasconi L, Mueller B, Christ-Crain M (2019) Influence of Outdoor Temperature and Relative Humidity on Incidence and Etiology of Hyponatremia. J Clin Endocrinol Metab 104:1304-1312. https://doi.org/10.1210/jc.2018-01507
395. Salahudeen AK, Ali N, George M, Lahoti A, Palla S (2014) Tolvaptan in hospitalized cancer patients with hyponatremia: a double-blind, randomized, placebo-controlled clinical trial on efficacy and safety. Cancer 120:744-51. https://doi.org/10.1002/cncr.28468
396. Saleem S, Yousuf I, Gul A, Gupta S, Verma S (2014) Hyponatremia in stroke. Ann Indian Acad Neurol 17:55-7. https://doi.org/10.4103/0972-2327.128554
397. Salterain-Gonzalez N, Esteban-Fernández A, García-López M, Lavilla-Royo FJ, Gavira-Gómez JJ (2013) Efficacy of tolvaptan in patients hospitalized for heart failure with refractory hyponatremia. Clinical experience in daily practice. Rev Esp Cardiol (Engl Ed) 66:503-4. https://doi.org/10.1016/j.rec.2012.12.015
398. Saramma P, Menon RG, Srivastava A, Sarma PS (2013) Hyponatremia after aneurysmal subarachnoid hemorrhage: Implications and outcomes. J Neurosci Rural Pract 4:24-8. https://doi.org/10.4103/0976-3147.105605
399. Sato N, Gheorghiade M, Kajimoto K, Munakata R, Minami Y, Mizuno M, Aokage T, Asai K, Sakata Y, Yumino D, Mizuno K, Takano T; ATTEND Investigators (2013) Hyponatremia and in-hospital mortality in patients admitted for heart failure (from the ATTEND registry). Am J Cardiol 111:1019-25. https://doi.org/10.1016/j.amjcard.2012.12.019
400. Sato Y, Yoshihisa A, Oikawa M, Nagai T, Yoshikawa T, Saito Y, Yamamoto K, Takeishi Y, Anzai T (2019) Hyponatremia at discharge is associated with adverse prognosis in acute heart failure syndromes with preserved ejection fraction: a report from the JASPER registry. Eur Heart J Acute Cardiovasc Care 8:623-633. https://doi.org/10.1177/2048872618822459
401. Schack LH, Mouritsen LS, Elowsson C, Krarup-Hansen A, Safwat A (2015) The Danish experience with trabectedin treatment for metastatic sarcoma: Importance of hyponatremia. Acta Oncol 54:34-40. https://doi.org/10.3109/0284186X.2014.958530
402. Schiara LAM, Moirano G, Grosso E, Richiardi L, Tibaldi M, Spertino E, Vezza C, Isaia GC, Massaia M, D'Amelio P (2020) Hyponatremia, Hypokalemia, and Fragility Fractures in Old Patients: More than an Association? Calcif Tissue Int 106:599-607. https://doi.org/10.1007/s00223-020-00675-6
403. Schuetz P, Haubitz S, Christ-Crain M, Albrich WC, Zimmerli W, Mueller B; ProHOSP Study Group (2013) Hyponatremia and anti-diuretic hormone in Legionnaires'disease. BMC Infect Dis 13:585. https://doi.org/10.1186/1471-2334-13-585
404. Schur S, Lasry O, Tewfik MA, Di Maio S (2020) Assessing the association of tumor consistency and gland manipulation on hormonal outcomes and delayed hyponatremia in pituitary macroadenoma surgery. Interdiscip. Neurosurg
405. Seal AD, Anastasiou CA, Skenderi KP, Echegaray M, Yiannakouris N, Tsekouras YE, Matalas AL, Yannakoulia M, Pechlivani F, Kavouras SA (2019) Incidence of Hyponatremia During a Continuous 246-km Ultramarathon Running Race. Front Nutr 6:161. https://doi.org/10.3389/fnut.2019.00161
406. See AP, Wu KC, Lai PM, Gross BA, Du R (2016) Risk factors for hyponatremia in aneurysmal subarachnoid hemorrhage. J Clin Neurosci 32:115-8. https://doi.org/10.1016/j.jocn.2016.04.006
407. Selmer C, Madsen JC, Torp-Pedersen C, Gislason GH, Faber J (2016) Hyponatremia, all-cause mortality, and risk of cancer diagnoses in the primary care setting: A large population study. Eur J Intern Med 36:36-43. https://doi.org/10.1016/j.ejim.2016.07.028
408. Shah A, Sabir S, Artani M, Salam O, Khan S, Rizwan A (2019) Significance of Hyponatremia as an Independent Factor in Predicting Short-term Mortality in Patients with Hemorrhagic Stroke. Cureus 11:e4549. https://doi.org/10.7759/cureus.4549
409. Shanmugam E, Doss CR, George M, Jena A, Rajaram M, Ramaraj B, Anjaneyan K, Kanagesh B (2016) Effect of tolvaptan on acute heart failure with hyponatremia--a randomized, double blind, controlled clinical trial. Indian Heart J 68 Suppl 1(Suppl 1):S15-21. https://doi.org/10.1016/j.ihj.2015.07.006
410. Sharif S, Dominguez M, Imbriano L, Mattana J, Maesaka JK (2015) Recognition of Hyponatremia As a Risk Factor for Hip Fractures in Older Persons. J Am Geriatr Soc 63:1962-4. https://doi.org/10.1111/jgs.13619
411. Sharma AK, Vegh EM, Kandala J, Orencole M, Januszkiewicz L, Bose A, Miller A, Parks KA, Heist EK, Singh JP (2014) Usefulness of hyponatremia as a predictor for adverse events in patients with heart failure receiving cardiac resynchronization therapy. Am J Cardiol 114:83-7. https://doi.org/10.1016/j.amjcard.2014.04.009
412. Shavit L, Merin O, Grenader T, Jacobson E, Waldenberg C, Bitran D, Fink D, Silberman S (2018) Hyponatremia Predicts Poor Outcomes in Patients With Chronic Kidney Disease Undergoing Heart Operation. Ann Thorac Surg 106:696-701. https://doi.org/10.1016/j.athoracsur.2018.04.015
413. Shavit L, Mikeladze I, Torem C, Slotki I (2014) Mild hyponatremia is associated with functional and cognitive decline in chronic hemodialysis patients. Clin Nephrol 82:313-9. https://doi.org/10.5414/CN108335
414. Shchekochikhin DY, Schrier RW, Lindenfeld J, Price LL, Jaber BL, Madias NE (2013) Outcome differences in community- versus hospital-acquired hyponatremia in patients with a diagnosis of heart failure. Circ Heart Fail 6:379-86. https://doi.org/10.1161/CIRCHEARTFAILURE.112.000106
415. Shimada A, Takeuchi H, Fukuda K, Suda K, Nakamura R, Wada N, Kawakubo H, Kitagawa Y (2018) Hyponatremia in patients with esophageal cancer treated with chemotherapy including cisplatin. Esophagus 15:209-216. https://doi.org/10.1007/s10388-018-0615-y
416. Shirai Y, Miura K, Shimizu S, Hattori M, Shimizu N (2019) Risk factors for hyponatremia after hypotonic fluid infusion. Pediatr Int 61:1239-1243. https://doi.org/10.1111/ped.14000
417. Shoaf SE, Bricmont P, Dandurand A (2017) Low-dose tolvaptan PK/PD: comparison of patients with hyponatremia due to syndrome of inappropriate antidiuretic hormone secretion to healthy adults. Eur J Clin Pharmacol 73:1399-1408. https://doi.org/10.1007/s00228-017-2302-7
418. Sidrat-ul-Muntaha, Sehrish, Farqaleet M (2018) Association of decompensated liver cirrhosis patient's mortality rate in the presence or absence of hyponatremia and meld score (11 - 20). Indo Am. J. Pharm. Sci 5:9039-9043
419. Sigal SH, Amin A, Chiodo JA 3rd, Sanyal A (2018) Management Strategies and Outcomes for Hyponatremia in Cirrhosis in the Hyponatremia Registry. Can J Gastroenterol Hepatol 2018:1579508. https://doi.org/10.1155/2018/1579508
420. Sim JK, Ko RE, Na SJ, Suh GY, Jeon K (2020) Intensive care unit-acquired hyponatremia in critically ill medical patients. J Transl Med 18:268. https://doi.org/10.1186/s12967-020-02443-4
421. Singh A, Ahuja R, Sethi R, Pradhan A, Srivastava V (2019) Prevalence and incidence of hyponatremia and their association with diuretic therapy: Results from North India. J Family Med Prim Care 8:3925-3930. https://doi.org/10.4103/jfmpc.jfmpc_604_19
422. Singh N, Tai JY, Dimech J, Gormack NJ, Cameron AJD, Lightfoot NJ (2020) Predictors of hyponatremia following elective primary unilateral knee arthroplasty at a tertiary centre: A retrospective observational cohort and predictive model. J Orthop 21:491-495. https://doi.org/10.1016/j.jor.2020.09.004
423. Sinno E, De Meo D, Cavallo AU, Petriello L, Ferraro D, Fornara G, Persiani P, Villani C (2020) Is postoperative hyponatremia a real threat for total hip and knee arthroplasty surgery? Medicine (Baltimore) 99:e20365. https://doi.org/10.1097/MD.0000000000020365
424. Sohail SS, Mughal N, Khan S (2018) Study to know the prevalence of hyponatremia in patients of hepatic encephalopathy having chronic liver disease. Indo Am. J. Pharm. Sci 5:14161-14165
425. Soiza RL, Cumming K, Clark AB, Bettencourt-Silva JH, Metcalf AK, Bowles KM, Potter JF, Myint PK (2015) Hyponatremia predicts mortality after stroke. Int J Stroke 10 Suppl A100:50-5. https://doi.org/10.1111/ijs.12564
426. Solak Y (2016) Comparison of serum sodium levels measured by blood gas analyzer and biochemistry autoanalyzer in patients with hyponatremia, eunatremia, and hypernatremia. Am J Emerg Med 34:1473-9. https://doi.org/10.1016/j.ajem.2016.04.037
427. Sood L, Sterns RH, Hix JK, Silver SM, Chen L (2013) Hypertonic saline and desmopressin: a simple strategy for safe correction of severe hyponatremia. Am J Kidney Dis 61:571-8. https://doi.org/10.1053/j.ajkd.2012.11.032
428. Sood N, Sharma KN, Himral P, Sharma T, Kapoor D. Clinical profile of patients with hyponatremia in a tertiary care hospital in the sub-Himalayan region (2020) J Family Med Prim Care 9:834-838. https://doi.org/10.4103/jfmpc.jfmpc_788_19
429. Stangl-Kremser J, Kramer G, Shariat SF (2019) Association of Hyponatremia With Survival in Patients With Castration-resistant Prostate Cancer: A Clinical Commentary. Clin Genitourin Cancer 17:e1188-e1192. https://doi.org/10.1016/j.clgc.2019.08.001
430. Sternal D, Wilczyński K, Szewieczek J (2016) Pressure ulcers in palliative ward patients: hyponatremia and low blood pressure as indicators of risk. Clin Interv Aging 12:37-44. https://doi.org/10.2147/CIA.S122464
431. Stieglmair S, Lindner G, Lassnigg A, Mouhieddine M, Hiesmayr M, Schwarz C (2013) Body salt and water balances in cardiothoracic surgery patients with intensive care unit-acquired hyponatremia. J Crit Care 28:1114.e1-5. https://doi.org/10.1016/j.jcrc.2013.05.017
432. Su Y, Ma M, Zhang H, Pan X, Zhang X, Zhang F, Lv Y, Yan C (2020) Prognostic value of serum hyponatremia for outcomes in patients with heart failure with preserved ejection fraction: An observational cohort study. Exp Ther Med 20:101. https://doi.org/10.3892/etm.2020.9231
433. Suárez V, Norello D, Sen E, Todorova P, Hackl MJ, Hüser C, Grundmann F, Kubacki T, Becker I, Peri A, Burst (2020) Impairment of Neurocognitive Functioning, Motor Performance, and Mood Stability in Hospitalized Patients With Euvolemic Moderate and Profound Hyponatremia. Am J Med 133:986-993.e5. https://doi.org/10.1016/j.amjmed.2019.12.056
434. Sugiyama Y, Naiki T, Tasaki Y, Kondo Y, Kataoka T, Etani T, Iida K, Nozaki S, Ando R, Osaga S, Yasui T, Kimura K (2020) Prognostic significance of hyponatremia induced by systemic chemotherapy in a hospital-based propensity score-matched analysis. Int J Clin Oncol 25:541-551. https://oi.org/10.1007/s10147-019-01579-8
435. Sun NH, Wang SH, Liu JN, Liu A, Gong WJ, Liu Y, Sun P, Li H (2017) The productions of atrial natriuretic peptide and arginine vasopressin in small cell lung cancer with brain metastases and their associations with hyponatremia. Eur Rev Med Pharmacol Sci 21:4104-4112
436. Tachi T, Yokoi T, Goto C, Umeda M, Noguchi Y, Yasuda M, Minamitani M, Mizui T, Tsuchiya T, Teramachi H (2015) Hyponatremia and hypokalemia as risk factors for falls. Eur J Clin Nutr 69:205-10. https://doi.org/10.1038/ejcn.2014.195
437. Tamizifar B, Kheiry S, Fereidoony F (2015) Hyponatremia and 30 days mortality of patients with acute pulmonary embolism. J Res Med Sci 20:777-81. https://doi.org/10.4103/1735-1995.168402
438. Tanaka R, Suzuki Y, Takumi Y, Iwao M, Sato Y, Hashinaga K, Hiramatsu K, Kadota JI, Itoh H (2016) A Retrospective Analysis of Risk Factors for Linezolid-Associated Hyponatremia in Japanese Patients. Biol Pharm Bull 39:1968-1973. https://doi.org/10.1248/bpb.b16-00418
439. Tandukar S, Kim C, Kalra K, Verma S, Palevsky PM, Puttarajappa C (2020) Severe Hyponatremia and Continuous Renal Replacement Therapy: Safety and Effectiveness of Low-Sodium Dialysate. Kidney Med 2:437-449. https://doi.org/10.1016/j.xkme.2020.05.007
440. Tasdemir V, Oguz AK, Sayın I, Ergun I (2015) Hyponatremia in the outpatient setting: clinical characteristics, risk factors, and outcome. Int Urol Nephrol 47:1977-83. https://doi.org/10.1007/s11255-015-1134-6
441. Thorpe O, Cuesta M, Fitzgerald C, Feely O, Tormey WP, Sherlock M, Williams DJ, Thompson CJ, Garrahy A (2021) Active management of hyponatraemia and mortality in older hospitalised patients compared with younger patients: results of a prospective cohort study. Age Ageing 50:1144-1150. https://doi.org/10.1093/ageing/afaa248
442. Tiseo M, Buti S, Boni L, Mattioni R, Ardizzoni A (2014) Prognostic role of hyponatremia in 564 small cell lung cancer patients treated with topotecan. Lung Cancer 86:91-5. https://doi.org/10.1016/j.lungcan.2014.07.022
443. Tobin G, Chacko AG, Simon R (2018) Evaluation of NT-ProBNP as a marker of the volume status of neurosurgical patients developing hyponatremia and natriuresis: A pilot study. Neurol India 66:1383-1388. https://doi.org/10.4103/0028-3886.241401
444. Tokgöz Akyil F, Akyil M, Çoban Ağca M, Güngör A, Ozantürk E, Söğüt G, Alparslan Bekir S, Topbaş A, Türker H, Sevim T (2019) Hyponatremia prolongs hospital stay and hypernatremia better predicts mortality than hyponatremia in hospitalized patients with community-acquired pneumonia. Tuberk Toraks 67:239-247. https://doi.org/10.5578/tt.68779
445. Tominaga N, Fernandez SJ, Mete M, Shara NM, Verbalis JG (2018) Hyponatremia and the risk of kidney stones: A matched case-control study in a large U.S. health system. PLoS One 13:e0203942. https://doi.org/10.1371/journal.pone.0203942
446. Tominaga N, Kida K, Inomata T, Sato N, Izumi T, Akashi YJ, Shibagaki Y (2017) Effects of Tolvaptan Addition to Furosemide in Normo- and Hyponatremia Patients with Heart Failure and Chronic Kidney Disease Stages G3b-5: A Subanalysis of the K-STAR Study. Am J Nephrol 46:417-426. https://doi.org/10.1159/000481995
447. Tomita Y, Kurozumi K, Inagaki K, Kameda M, Ishida J, Yasuhara T, Ichikawa T, Sonoda T, Otsuka F, Date I (2019) Delayed postoperative hyponatremia after endoscopic transsphenoidal surgery for pituitary adenoma. Acta Neurochir (Wien) 161:707-715. https://doi.org/10.1007/s00701-019-03818-3
448. Topaz G, Pereg D, Gur E, Kitay-Cohen Y, Ben-Zvi E, Eitan M, Benchetrit S, Cohen-Hagai K (2020) Hyponatremia is associated with poor prognosis among patients with chest pain discharged from internal medicine wards following acute coronary syndrome-rule-out. Coron Artery Dis 31:147-151. https://doi.org/10.1097/MCA.0000000000000786
449. Tosh P, Rajan S, Kadapamannil D, et al (2017) Efficacy of oral tolvaptan versus 3% hypertonic saline for correction of hyponatraemia in post-operative patients. Indian J Anaesth 61:996–1001. https://doi.org/10.4103/ija.IJA_581_17
450. Trifanescu RA, Soare D, Cirstoiu C, Popescu G, Pascu AM, Poroch V, Toma S, Poiana C (2018) Mild Chronic Hyponatremia and Osteoporctic Fractures Risk in Elderly. Rev. Chim 69:3520-3523
451. Tsapepas D, Chiles M, Babayev R, Rao MK, Jaitly M, Salerno D, Mohan S (2016) Incidence of Hyponatremia with High-Dose Trimethoprim-Sulfamethoxazole Exposure. Am J Med 129:1322-1328. https://doi.org/10.1016/j.amjmed.2016.07.012
452. Tseng MH, Cheng CJ, Sung CC, Chou YC, Chu P, Chen GS, Lin SH (2014) Hyponatremia is a surrogate marker of poor outcome in peritoneal dialysis-related peritonitis. BMC Nephrol 15:113. https://doi.org/10.1186/1471-2369-15-113
453. Turgutalp K, Ozhan O, Gok Oguz E, Horoz M, Camsari A, Yilmaz A, Kiykim A, Arici M (2013) Clinical features, outcome and cost of hyponatremia-associated admission and hospitalization in elderly and very elderly patients: a single-center experience in Turkey. Int Urol Nephrol 45:265-73. https://doi.org/10.1007/s11255-012-0307-9
454. Umbrello M, Mantovani ES, Formenti P, Casiraghi C, Ottolina D, Taverna M, Pezzi A, Mistraletti G, Iapichino G (2016) Tolvaptan for hyponatremia with preserved sodium pool in critically ill patients. Ann Intensive Care 6:1. https://doi.org/10.1186/s13613-015-0096-2
455. Usala RL, Fernandez SJ, Mete M, Cowen L, Shara NM, Barsony J, Verbalis JG (2015) Hyponatremia Is Associated With Increased Osteoporosis and Bone Fractures in a Large US Health System Population. J Clin Endocrinol Metab 100:3021-31. https://doi.org/10.1210/jc.2015-1261
456. Usala RL, Fernandez SJ, Mete M, Shara NM, Verbalis JG (2019) Hyponatremia Is Associated With Increased Osteoporosis and Bone Fractures in Patients With Diabetes With Matched Glycemic Control. J Endocr Soc 3:411-426. https://doi.org/10.1210/js.2018-00320
457. Uyar S, Dolu S, Tasi Z, Babacan Abanonu G, Gurler MY, Gorar S, Sahinturk Y, Bostan F, Cekin AH (2016) Evaluation of elderly patients hospitalized for hyponatremia: is hyponatremia a real independent risk factor affecting mortality in these patients?. Turk. J. Geriatr 19:139-145
458. Vallabhajosyula S, Varma MD, Vallabhajosyula S, Vallabhajosyula S (2016) Association of hyponatremia with in-hospital outcomes in infective endocarditis: A 5-year review from an Indian Intensive Care Unit. Indian J Crit Care Med 20:597-600. https://doi.org/10.4103/0972-5229.192051
459. Van Blijderveen JC, Straus SM, Rodenburg EM, Zietse R, Stricker BH, Sturkenboom MC, Verhamme KM (2014) Risk of hyponatremia with diuretics: chlorthalidone versus hydrochlorothiazide. Am J Med 127:763-71. https://doi.org/10.1016/j.amjmed
460. Van Houte J, Bindels AJ, Houterman S, Dong PV, den Ouden M, de Bock NE, Verberkmoes NJ, Curvers J, Bouwman AR (2021) Acute isotonic hyponatremia after single dose histidine-tryptophan-ketoglutarate cardioplegia: an observational study. Perfusion 36:440-446. https://doi.org/10.1177/0267659120946952
461. Van Wart SA, Shoaf SE, Mallikaarjun S, Mager DE (2013) Population pharmacokinetics of tolvaptan in healthy subjects and patients with hyponatremia secondary to congestive heart failure or hepatic cirrhosis. Biopharm Drug Dispos 34:336-47. https://doi.org/10.1002/bdd.1849
462. Vandergheynst F, Gombeir Y, Bellante F, Perrotta G, Remiche G, Mélot C, Mavroudakis N, Decaux G (2016) Impact of hyponatremia on nerve conduction and muscle strength. Eur J Clin Invest 46:328-33. https://doi.org/10.1111/eci.12597
463. Vannucci L, Parenti G, Simontacchi G, Rastrelli G, Giuliani C, Ognibene A, Peri A (2017) Hypothyroidism and hyponatremia: data from a series of patients with iatrogenic acute hypothyroidism undergoing radioactive iodine therapy after total thyroidectomy for thyroid cancer. J Endocrinol Invest 40:49-54. https://doi.org/10.1007/s40618-016-0525-6
464. Velat I, Bušić Ž, Jurić Paić M, Čulić V (2020) Furosemide and spironolactone doses and hyponatremia in patients with heart failure. BMC Pharmacol Toxicol 21:57. https://doi.org/10.1186/s40360-020-00431-4
465. Verbalis JG, Ellison H, Hobart M, Krasa H, Ouyang J, Czerwiec FS; Investigation of the Neurocognitive Impact of Sodium Improvement in Geriatric Hyponatremia: Efficacy and Safety of Tolvaptan (INSIGHT) Investigators (2016) Tolvaptan and Neurocognitive Function in Mild to Moderate Chronic Hyponatremia: A Randomized Trial (INSIGHT). Am J Kidney Dis 67:893-901. https://doi.org/10.1053/j.ajkd.2015.12.024
466. Verbrugge FH, Grodin JL, Mullens W, Taylor DO, Starling RC, Tang WH (2016) Transient Hyponatremia During Hospitalization for Acute Heart Failure. Am J Med 129:620-7. https://doi.org/10.1016/j.amjmed.2016.01.016
467. Verghese SC, Mahajan A, Uppal B (2019) Chronic versus New-Onset Hyponatremia in Geriatric Patients Undergoing Orthopedic Surgery. Int J Appl Basic Med Res 9:37-43. https://doi.org/10.4103/ijabmr.IJABMR_374_18
468. Vilapurathu JK, Rajarajan S (2014) A prospective study to compare the clinical efficacy of Tolvaptan with 3% hypertonic saline solution in hospitalized patients having hyponatremia. J Res Pharm Pract 3:34-6. https://doi.org/10.4103/2279-042X.132710
469. Voglis S, van Niftrik CHB, Staartjes VE, Brandi G, Tschopp O, Regli L, Serra C (2020) Feasibility of machine learning based predictive modelling of postoperative hyponatremia after pituitary surgery. Pituitary 23:543-551. https://doi.org/10.1007/s11102-020-01056-w
470. Wang CH, Tsai CE, Cheng KW, Chen CL, Huang CJ, Wu SC, Shih TH, Yang SC, Juang SE, Liu CK, Jawan B, Hou SY (2020) Anesthetic Fluid Management of Patients With Hyponatremia Undergoing Living Donor Liver Transplantation. Transplant Proc 52:1798-1801. https://doi.org/10.1016/j.transproceed.2020.01.144
471. Wang S, Zhang X, Han T, Xie W, Li Y, Ma H, Liebe R, Weng H, Ding HG (2018) Tolvaptan treatment improves survival of cirrhotic patients with ascites and hyponatremia. BMC Gastroenterol 18:137. https://doi.org/10.1186/s12876-018-0857-0
472. Wang WX, Song ZB, Zhang YP (2016) Hyponatremia in small cell lung cancer is associated with a poorer prognosis. Transl. Cancer Res 5:36-43
473. Wang Y, Liu J (2015) Hyponatremia is a predictor for poor outcome in Guillain-Barré syndrome. Neurol Res 37:347-51. https://doi.org/10.1179/1743132814Y.0000000455
474. Wannamethee SG, Shaper AG, Lennon L, Papacosta O, Whincup P (2016) Mild hyponatremia, hypernatremia and incident cardiovascular disease and mortality in older men: A population-based cohort study. Nutr Metab Cardiovasc Dis 26:12-9. https://doi.org/10.1016/j.numecd.2015.07.008
475. Ward FL, Tobe SW, Naimark DMJ (2018) The Role of Desmopressin in the Management of Severe, Hypovolemic Hyponatremia: A Single-Center, Comparative Analysis. Can J Kidney Health Dis 5:2054358118761051. https://doi.org/10.1177/2054358118761051
476. Watson H, Guevara M, Vilstrup H, Ginès P (2019) Improvement of hyponatremia in cirrhosis is associated with improved complex information processing. J Gastroenterol Hepatol 34:1999-2003. https://doi.org/10.1111/jgh.14683
477. Weir MA, Fleet JL, Vinden C, Shariff SZ, Liu K, Song H, Jain AK, Gandhi S, Clark WF, Garg AX (2014) Hyponatremia and sodium picosulfate bowel preparations in older adults. Am J Gastroenterol 109:686-94. https://doi.org/10.1038/ajg.2014.20
478. Wen Y, Zhou Y, Wang W, Wang Y, Lu X, Sun C, Liu P (2014) Characteristics of persistent hyponatremia and tolvaptan treatment in nine hospitalized patients with advanced HIV disease. HIV Clin Trials 15:126-32. https://doi.org/10.1310/hct1503-126
479. Winata AS, Jen WY, Teng ML, Hing WC, Iyer SG, Ma V, Chua HR (2019) Intravenous maintenance fluid tonicity and hyponatremia after major surgery- a cohort study. Int J Surg 67:1-7. https://doi.org/10.1016/j.ijsu.2019.04.019
480. Winograd D, Staggers KA, Sebastian S, Takashima M, Yoshor D, Samson SL (2020) An Effective and Practical Fluid Restriction Protocol to Decrease the Risk of Hyponatremia and Readmissions After Transsphenoidal Surgery. Neurosurgery 87:761-769. https://doi.org/10.1093/neuros/nyz555
481. Winther JA, Brynildsen J, Høiseth AD, Følling I, Brekke PH, Christensen G, Hagve TA, Verbalis JG, Omland T, Røsjø H (2016) Prevalence and Prognostic Significance of Hyponatremia in Patients with Acute Exacerbation of Chronic Obstructive Pulmonary Disease: Data from the Akershus Cardiac Examination (ACE) 2 Study. PLoS One 11:e0161232. https://doi.org/10.1371/journal.pone.0161232
482. Winzeler B, Jeanloz N, Nigro N, Suter-Widmer I, Schuetz P, Arici B, Bally M, Blum C, Bock A, Huber A, Mueller B, Christ-Crain M (2016) Long-term outcome of profound hyponatremia: a prospective 12 months follow-up study. Eur J Endocrinol 175:499-507. https://doi.org/10.1530/EJE-16-0500
483. Wolf P, Beiglböck H, Smaijs S, Wrba T, Rasoul-Rockenschaub S, Marculescu R, Gessl A, Luger A, Winhofer Y, Krebs M (2017) Hypothyroidism and Hyponatremia: Rather Coincidence Than Causality. Thyroid 27:611-615. https://doi.org/10.1089/thy.2016.0597
484. Woodfine JD, Sood MM, MacMillan TE, Cavalcanti RB, van Walraven C (2019) Derivation and Validation of a Novel Risk Score to Predict Overcorrection of Severe Hyponatremia: The Severe Hyponatremia Overcorrection Risk (SHOR) Score. Clin J Am Soc Nephrol 14:975-982. https://doi.org/10.2215/CJN.12251018
485. Woudstra J, de Boer MP, Hempenius L, van Roon EN (2020) Urea for hyponatraemia due to the syndrome of inappropriate antidiuretic hormone secretion. Neth J Med 78:125-131
486. Wozniak PA, Oledzka-Oreziak M, Ponialowska R, Motyl R, Wardyn KA (2017) Reduction in the volume of the front part of the hippocampus in schizophrenics with hyponatremia on hospital admission. Postep. Psychiatr. Neurol 26:63-74
487. Xu J, Chen X, Wang X, Zhu C, Hu Y, Yang X, Xu C, Shen X (2019) Preoperative Hyponatremia And Hypocalcemia Predict Poor Prognosis In Elderly Gastric Cancer Patients. Cancer Manag Res 11:8765-8780. https://doi.org/10.2147/CMAR.S211603
488. Xu L, Ye H, Huang F, Yang Z, Zhu B, Xu Y, Qiu Y, Li L (2014) Moderate/Severe hyponatremia increases the risk of death among hospitalized Chinese human immunodeficiency virus/acquired immunodeficiency syndrome patients. PLoS One 9:e111077. https://doi.org/10.1371/journal.pone.0111077
489. Xu R, Pi HC, Xiong ZY, Liao JL, Hao L, Liu GL, Ren YP, Wang Q, Zheng ZX, Duan LP, Dong J (2015) Hyponatremia and Cognitive Impairment in Patients Treated with Peritoneal Dialysis. Clin J Am Soc Nephrol 10:1806-13. https://doi.org/10.2215/CJN.02240215
490. Xu X, Lin S, Yang Y, Chen Y, Liu B, Li B, Wu Y, Meng F, Zhu Q, Li Y, Tang S, Yuan S, Shao L, Qi X (2020) Development of hyponatremia after terlipressin in cirrhotic patients with acute gastrointestinal bleeding: a retrospective multicenter observational study. Expert Opin Drug Saf 19:641-647. https://doi.org/10.1080/14740338.2020.1734558
491. Yamamoto Y, Takahashi Y, Imai K, Ohta A, Kagawa Y, Inoue Y (2019) Prevalence and risk factors for hyponatremia in adult epilepsy patients: Large-scale cross-sectional cohort study. Seizure 73:26-30. https://doi.org/10.1016/j.seizure.2019.10.013
492. Yamany A, Behiry ME, Ahmed SA (2020) Hyponatremia as an Inflammatory Marker of Lupus Activity Is a Fact or Fad: A Cross-Sectional Study. Open Access Rheumatol 12:29-34. https://doi.org/10.2147/OARRR.S237168
493. Yamazoe M, Mizuno A, Kohsaka S, Shiraishi Y, Kohno T, Goda A, Higuchi S, Yagawa M, Nagatomo Y, Yoshikawa T; West Tokyo Heart Failure Registry Investigators Tokyo, Japan (2018) Incidence of hospital-acquired hyponatremia by the dose and type of diuretics among patients with acute heart failure and its association with long-term outcomes. J Cardiol 71:550-556. https://doi.org/10.1016/j.jjcc.2017.09.015
494. Yan MT, Cheng CJ, Wang HY, Yang CS, Peng SJ, Lin SH (2016) Evaluating Hyponatremia in Non-Diabetic Uremic Patients on Peritoneal Dialysis. Perit Dial Int 36:196-204. https://doi.org/10.3747/pdi.2014.00239
495. Yang C, Wang G, Xu S, Li G, Wang Q (2020) Influence of early extensive posterior decompression on hyponatremia and cardiopulmonary dysfunction after severe traumatic cervical spinal cord injury: A clinical observational study. Medicine (Baltimore) 99:e21188. https://doi.org/10.1097/MD.0000000000021188
496. Yang HJ, Cheng WJ (2017) Antipsychotic use is a risk factor for hyponatremia in patients with schizophrenia: a 15-year follow-up study. Psychopharmacology (Berl) 234:869-876. https://doi.org/10.1007/s00213-017-4525-9
497. Yang LJ, Wu PH, Huang TH, Lin MY, Tsai JC (2018) Thiazide-associated hyponatremia attenuates the fracture-protective effect of thiazide: A population-based study. PLoS One 13:e0208712. https://doi.org/10.1371/journal.pone.0208712
498. Yang SM, Choi SN, Yu JH, Yoon HK, Kim WH, Jung CW, Suh KS, Lee KH (2018) Intraoperative hyponatremia is an independent predictor of one-year mortality after liver transplantation. Sci Rep 8:18023. https://doi.org/10.1038/s41598-018-37006-7
499. Yang Y, Sun N, Sun P, Zhang L (2017) Clinical Characteristics and Prognosis of Elderly Small Cell Lung Cancer Patients Complicated with Hyponatremia: A Retrospective Analysis. Anticancer Res 37:4681-4686. https://doi.org/10.21873/anticanres.11872
500. Yeh HC, Li CC, Wen SC, Singla N, Woldu SL, Robyak H, Huang CN, Ke HL, Li WM, Lee HY, Li CY, Yeh BW, Yang SF, Tu HP, Shariat SF, Sagalowsky AI, Raman JD, Lotan Y, Hsieh JT, Margulis V, Wu WJ (2020) Validation of Hyponatremia as a Prognostic Predictor in Multiregional Upper Tract Urothelial Carcinoma. J Clin Med 9:1218. https://doi.org/10.3390/jcm9041218
501. Yim SY, Seo YS, Jung CH, Kim TH, Kim ES, Keum B, Kim JH, An H, Yim HJ, Yeon JE, Jeen YT, Lee HS, Chun HJ, Byun KS, Um SH, Kim CD, Ryu HS (2015) Risk Factors for Developing Hyponatremia During Terlipressin Treatment: A Retrospective Analyses in Variceal Bleeding. J Clin Gastroenterol 49:607-12. https://doi.org/10.1097/MCG.0000000000000217
502. Yoo BS, Park JJ, Choi DJ, Kang SM, Hwang JJ, Lin SJ, Wen MS, Zhang J, Ge J, COAST investigators (2015) Prognostic value of hyponatremia in heart failure patients: an analysis of the Clinical Characteristics and Outcomes in the Relation with Serum Sodium Level in Asian Patients Hospitalized for Heart Failure (COAST) study. Korean J Intern Med 30:460-70. https://doi.org/10.3904/kjim.2015.30.4.460.
503. Yoon HK, Lee HC, Kim YH, Lim YJ, Park HP (2019) Predictive Factors for Delayed Hyponatremia After Endoscopic Transsphenoidal Surgery in Patients with Nonfunctioning Pituitary Tumors: A Retrospective Observational Study. World Neurosurg 122:e1457-e1464. https://doi.org/10.1016/j.wneu.2018.11.085
504. Yoon J, Ahn SH, Lee YJ, Kim CM (2015) Hyponatremia as an independent prognostic factor in patients with terminal cancer. Support Care Cancer 23:1735-40. https://doi.org/10.1007/s00520-014-2522-7
505. Yoshioka K, Matsue Y, Kagiyama N, Yoshida K, Kume T, Okura H, Suzuki M, Matsumura A, Yoshida K, Hashimoto Y (2016) Recovery from hyponatremia in acute phase is associated with better in-hospital mortality rate in acute heart failure syndrome. J Cardiol 67:406-11. https://doi.org/10.1016/j.jjcc.2015.12.004
506. Yumoto T, Sato K, Ugawa T, Ichiba S, Ujike Y (2015) Prevalence, risk factors, and short-term consequences of traumatic brain injury-associated hyponatremia. Acta Med Okayama 69:213-8. https://doi.org/10.18926/AMO/53557
507. Zhang G, Lian R, Sun L, Liu H, Wang Y, Zhou L (2020) Redefined hyponatremia as a marker to exclude the diagnosis of anastomotic leakage after colorectal cancer surgery. J Int Med Res 48:300060520950565. https://doi.org/10.1177/0300060520950565
508. Zhang X, Li XY (2020) Prevalence of hyponatremia among older inpatients in a general hospital. Eur Geriatr Med 11:685-692. https://doi.org/10.1007/s41999-020-00320-3
509. Zhou C, Zhang N, Wang Y, He TT, Zhou K, Xiao XH, Li J, Gong M (2019) Hyponatremia is an independent risk factor for mortality from hepatitis B virus-associated acute-on-chronic liver failure. Int. J. Clin. Exp. Med 12:2683-2689
510. Zieschang T, Wolf M, Vellappallil T, Uhlmann L, Oster P, Kopf D (2016) The Association of Hyponatremia, Risk of Confusional State, and Mortality. Dtsch Arztebl Int 16113:855-862. https://doi.org/10.3238/arztebl.2016.0855
511. Zobel MJ, Stewart L (2020) Hyponatremia is associated with more severe biliary disease. World J Gastrointest Surg 12:45-54. https://doi.org/10.4240/wjgs.v12.i2.45
